# Organocatalyzed Atom Transfer Radical (Co)Polymerization of Fluorinated and POSS-Containing Methacrylates: Synthesis and Properties of Linear and Star-Shaped (Co)Polymers

**DOI:** 10.3390/polym18010141

**Published:** 2026-01-04

**Authors:** Hleb Baravoi, Heorhi Belavusau, Aliaksei Vaitusionak, Valeriya Kukanova, Anastasia Frolova, Peter Timashev, Hongzhi Liu, Sergei Kostjuk

**Affiliations:** 1 Research Institute for Physical Chemical Problems of the Belarusian State University, 14 Leningradskaya St., 220006 Minsk, Belarus; glebborovoj268@gmail.com (H.B.); george.vusau@gmail.com (H.B.); aliaksei.vaitusionak@gmail.com (A.V.); 2 Department of Chemistry, Belarusian State University, 14 Leningradskaya St., 220006 Minsk, Belarus; 3 Institute for Regenerative Medicine, Sechenov First Moscow State Medical University, 8-2 Trubetskaya St., 119991 Moscow, Russia; kukanova_v_s@staff.sechenov.ru (V.K.); frolova_a_a_2@staff.sechenov.ru (A.F.); timashev_p_s@staff.sechenov.ru (P.T.); 4 School of Chemistry and Chemical Engineering, Shandong University, Jinan 250100, China; liuhongzhi@sdu.edu.cn; 5 Equipe Chimie des Polymeres, Institut Parisien de Chimie Moleculaire, Sorbonne Universite, CNRS, 4 Place Jussieu, CEDEX 05, 75252 Paris, France

**Keywords:** O-ATRP, fluorinated coatings, POSS, visible light induced polymerization, star-shaped copolymers, C-F photoactivation

## Abstract

Hybrid fluorinated copolymers containing POSS moieties along with fluorinated homopolymers were synthesized via organocatalyzed atom transfer radical (co)polymerization (O-ATRP) of fluoroalkyl methacrylate (**FMA**) and a POSS-based monomer (**IBSS**) using perylene as a photocatalyst. Linear and four- and eight-armed star-shaped (co)polymers in a wide range of molecular weights with M_n_(SEC) up to 53,100 g/mol for poly(**FMA**), 22,700 g/mol for poly(**IBSS**) and 87,300 g/mol for poly(**FMA***-co-***IBSS**) were successfully prepared. During polymerization, C–F activation was found to induce chain transfer and branching reactions, contributing to structural diversity. A mechanism for chain transfer to the polymer resulting in branching was proposed, applying density functional theory (DFT). Films based on the obtained (co)polymers showed tunable morphology, high thermal stability (up to 306 °C) and hydrophobicity, with water contact angles reaching 98°.

## 1. Introduction

Hydrophobic surfaces are widely used in waterproofing, antifouling, anti-corrosion, anti-frosting, self-cleaning and oil–water separation applications due to their unique wetting properties [1,2,3]. The two main requirements for obtaining a hydrophobic surface are low surface energy and suitable roughness. Fluoropolymers are highly valued for their exceptional combination of physical and chemical properties, which include low surface energy, oil and water repellency [4,5,6], low refractive index [7,8,9], low dielectric permittivity [10,11,12], high thermal and chemical stability [13,14,15], and resistance to flammability and moisture absorption [16,17,18,19,20]. These properties are primarily due to their strong, highly polar carbon–fluorine (C–F) bonds, which contribute to their outstanding durability and inertness to a wide range of harsh environmental and chemical conditions, including solvents, acids, alkalis and oxidative agents [16,17,18,19,20]. As a result, fluorinated polymers find widespread use in diverse industries, serving as critical components in coatings, emulsifiers, surfactants, electrical insulation materials, antifouling and protective paints, biomaterials, and lubricants [21,22,23,24]. The good copolymerizability of (meth)acrylate-type fluorinated monomers with a great variety of other (meth)acrylate monomers further broadens their applicability, allowing creation of advanced materials with tailored surface properties and enhanced performance in demanding environments [25,26].

On the other hand, polyhedral oligomeric silsesquioxane (POSS) is a unique nanoscale inorganic compound with a cage-like silica core measuring approximately 1–3 nm, surrounded by organic functional groups that enhance solubility and compatibility with polymers [27,28]. When incorporated into polymer matrices, POSS significantly improves their properties, such as mechanical strength, thermal stability, hydrophobicity and resistance to flammability, oxidation and chemical exposure [29,30]. Moreover, POSS units tend to migrate to the surface of polymer films, modifying surface roughness and morphology [29]. POSS can be used as a monomer, initiator and crosslinking agent in order to obtain POSS-containing materials with various architectures [31,32]. These hybrid polymers with different architectures, such as block copolymers, star-shaped polymers and dendrimers, have been synthesized by controlled/living radical polymerization techniques [28,33,34,35,36,37,38,39,40,41].

The combination of fluoropolymers with hybrid POSS fragments in a single structure significantly enhances material performance, enabling a wide range of applications. Films and coatings derived from these hybrid copolymers demonstrate outstanding thermal and chemical stability. However, there are only a limited number of studies focused on the synthesis of copolymers based on POSS and fluoroacrylates [42,43,44,45]. Particularly, linear and star-shaped POSS-based fluorinated (co)polymers were synthesized by means of ATRP using POSS-initiators, and such copolymers were used as hydrophobic porous films with controllable pore sizes [42,43]. Recently, Nakatani et al. [46] and Ponnupandian et al. [47] reported the preparation of block copolymers based on 2,2,2-trifluoroethyl methacrylate and either methacryloethyl POSS or methacryloisobutyl POSS via RAFT polymerization. Films produced from the synthesized block copolymers were characterized by high roughness (10.5 nm) and a high water contact angle (128°) [47].

Most of the studies on the synthesis of hydrophobic fluorinated polymers deal with metal-catalyzed ATRP [25,31,48]. While this provides precise control over molecular weight distribution, monomer sequence and chain-end functionality, its reliance on transition-metal complexes poses limitations in monomer scope and challenges, including catalyst removal from the final product and sensitivity to oxidation of lower-valent metal species [48,49]. In strong contrast, organocatalyzed ATRP (O-ATRP), which has recently emerged as an attractive alternative to classical metal-catalyzed ATRP, eliminates the need for metal catalysts by employing organic photoredox catalysts. Moreover, the use of light as an external stimulus offers spatiotemporal control over polymerization, enabling on-demand initiation, reversible switching, and precise regulation of chain growth [50,51]. These features make the development of efficient initiating systems for photopolymerization highly important, as they broaden the scope of ATRP-derived methodologies and open opportunities in advanced material design and sustainable polymer synthesis [52,53,54,55]. This technique has already been used for the controlled polymerization of a broad spectrum of monomers, especially of methacrylate-type ones [50], preserving all advantages of traditional ATRP, like high conversion, well-defined architectures and precisely tailored properties.

The objective of this study was to obtain a series of hybrid fluorinated polymers bearing POSS moieties with different architectures and compositions to establish the relationship between these parameters and the thermal and surface properties of the target materials. For this purpose, O-ATRP of **FMA** and **IBSS** has been investigated for the first time using perylene as a photocatalyst and different organic halides as initiators for the preparation of linear and star-shaped polymers and random copolymers. As a result, the proposed photoinitiating system was enabled to synthesize fluorinated homopolymers and POSS-based copolymers with linear and four- and eight-armed star-shaped architectures in a wide range of molecular weights with M_n_(SEC) up to 53,100 g/mol for poly(**FMA**) and up to 87,300 g/mol for poly(**FMA**-*co*-**IBSS**), respectively. The undesirable C-F activation process was clarified via computational chemistry, which allowed us to explain the mechanism of chain transfer to the polymer resulting in chain branching. Finally, it was demonstrated that the architecture and composition of (co)polymers influence film morphology, their thermal stability and their hydrophobicity.

## 2. Materials and Methods

### 2.1. Materials

N,N-Dimethylformamide (Sigma-Aldrich, ≥99%, St. Louis, MO, USA) was distilled twice under reduced pressure and dried over molecular sieves, 4 Å; toluene (Sigma-Aldrich, ≥99%, St. Louis, MO, USA) was distilled and dried over molecular sieves, 4 Å; ethyl α-bromophenylacetate (EBP) (Sigma-Aldrich, 97%, St. Louis, MO, USA), 2,2,3,4,4,4-hexafluorobutyl methacrylate (**FMA**) (TCI, >98%, Tokyo, Japan) and methyl methacrylate (MMA, TCI, >99%, Tokyo, Japan) were distilled under reduced pressure and stored under an argon atmosphere. Tetrahydrofuran (Sigma-Aldrich, ≥99%, St. Louis, MO, USA) was treated with KOH and distilled twice over Na under an inert atmosphere. Methacryloxypropyl-substituted poly(isobutyl-T_8_-silsesquioxane (IBSS) (Sigma-Aldrich, St. Louis, MO, USA), perylene (Per) (Sigma-Aldrich, for synthesis, St. Louis, MO, USA), CDCl_3_ (neoFroxx, 99.8%, Einhausen, Germany), dimethyl sulfoxide-d6 (Sigma-Aldrich, 99.9%, St. Louis, MO, USA) and methanol (Sigma-Aldrich, 99.9%, St. Louis, MO, USA) were used as received. Pentaerythritol tetrakis(2-bromoisobutyrate) (PETBiB) and an octafunctional POSS-based initiator (POSSBr_8_) were synthesized according to previously published methods [56,57], and their structures were confirmed via ^1^H NMR spectroscopy (Figure S1). 

### 2.2. Methods

^1^H (500 MHz) NMR spectra of polymers were recorded in CDCl_3_ or DMSO-d^6^ at 25 °C on a Brucker AC-500 spectrometer (Billerica, MA, USA) calibrated relative to the solvent peak. Differential scanning calorimetry (DSC) and thermogravimetric analysis (TGA) measurements were carried out using a Netzsch STA (simultaneous thermal analysis) 449 F3 device (Selb, Germany) at a heating rate of 20 °C min^−1^ under nitrogen flow. Size exclusion chromatography (SEC) was performed on an Ultimate 3000 Thermo Scientific (Sunnyvale, CA, USA) apparatus with an Agilent (Santa Clara, CA, USA) PLgel 5 μm MIXED-C (300 × 7.5 mm) column and one precolumn (PLgel 5 μm guard 50 × 7.5 mm) thermostated at 30 °C. The detection was achieved with a differential refractometer (Sunnyvale, CA, USA) (thermostated at 35 °C). Tetrahydrofuran (THF) was eluted at a flow rate of 1.0 mL min^−1^. The calculation of molecular weights and polydispersity was carried out using polystyrene standards (Polymer Labs, Germany).

Thin films (∼100–150 nm) of the hydrophobic homopolymers and copolymers were prepared using a WS-650MZ spin-coater (Laurell Technologies Corporation, Lansdale, PA, USA) to deposit 3 wt% solutions in tetrohydrofuran onto a glass substrate (ø30 mm). The centrifugation program took place in two stages, step 1–150 rpm for 10 s and step 2–4000 rpm for 30 s, at room temperature. The prepared films were dried overnight in a desiccator at room temperature and 10% humidity.

Morphological atomic force microscopy (AFM) studies of the surface were performed using an atomic force microscope (BioScope Resolve, Bruker, Billerica, MA, USA) combined with an Axio Observer inverted optical microscope (Carl Zeiss Microscopy GmbH, Jena, Germany). ScanAsyst Air (Bruker, Billerica, MA, USA) cantilevers with a nominal spring constant of 0.4 N/m, nominal frequency of 70 kHz and nominal tip radius of 2 nm were selected for scanning. The exact value of the spring constant was determined by the thermal tune method, and the exact value of the tip radius was determined using NanoScope Analysis tip-evaluation software (version: 1.50) after scanning the titanium roughness sample (Bruker, Billerica, MA, USA). AFM studies of the dried films were carried out at room temperature (25 °C) in PeakForce QNM mode. The ROIs of the films were 5 × 5, 10 × 10 and 100 × 100 µm scan sizes. Two to three AFM images of each size were obtained from the surface of each sample. NanoScope Analysis v1.9 software (Bruker, Billerica, MA, USA) was used to analyze the images.

The hydrophobicity of the surface of the homopolymers and copolymers was studied by measuring contact angles, which was carried out by the sessile drop method using an Acam-MSC01 device (Apex Instruments, Kolkata, India), with distilled water and vegetable oil being used as liquids. The temperatures of the instrument table and the liquid were 22–25 °C. The measurements were taken at least 3 times for each sample.

### 2.3. Polymerization Procedures

Synthesis of (co)polymers with various architectures was carried out under a dry argon atmosphere in a Schlenk tube equipped with a stirrer bar. The transfer of liquid reagents to a reactor was conducted via dry syringes in a continuous argon flow.

#### 2.3.1. Synthesis of FMA-Based Homopolymers with Various Architectures

In a typical polymerization experiment, a solution of an initiator in DMF (125 μL, 108 mM for EBP, 27 mM for PETBiB and 13.5 mM for POSSBr_8_) and a solution of a photocatalyst (Per) in DMF (125 μL, 12 mM) were sequentially added to FMA (250 μL, 1.35 mmol). After 3 freeze–pump–thaw cycles, polymerization was started by irradiation with a blue LED (wavelength 435 nm). Samples for kinetics experiments were withdrawn after a predetermined time and then were dissolved in CDCl_3_ for PFMA and DMSO-d^6^ for P(FMA)_4_ and P(FMA)_8_. Conversion of the monomer was calculated using NMR spectroscopy data. In order to separate polymers from the reaction mixture, they were precipitated in water, and then dissolved in THF and reprecipitated in n-hexane. Polymers were separated from the solution by centrifugation and dried in vacuum.

#### 2.3.2. Synthesis of IBSS-Based Homopolymers with Various Architectures

In a typical polymerization experiment, a solution of an initiator in toluene (100 μL, 212 mM for EBP and 53 mM for PETBiB) and a solution of a photocatalyst (Per) in toluene (100 μL, 2.35 mM) were sequentially added to solid **IBSS** (200 mg, 0.212 mmol). After 3 freeze–pump–thaw cycles, the reaction mixture was irradiated with a blue LED (wavelength 435 nm). Samples for kinetics experiments were withdrawn after a predetermined time and then were dissolved in CDCl_3_. Conversion of the monomer was calculated using NMR spectroscopy data. For purification, polymers were reprecipitated from chloroform to methanol. Polymers were separated from the solution by centrifugation and dried in vacuum.

#### 2.3.3. Synthesis of Random Copolymers Based on FMA and IBSS with Various Architectures

In a typical polymerization experiment, a solution of an initiator (52.5 μL, 128 mM for EBP, 32 mM for PETBiB and 16 mM for POSSBr_8_) in toluene and a solution of a photocatalyst (Per) in toluene (52.5 μL, 14 mM) were added sequentially to the monomer mixture (133.5 mg of IBSS (0.14 mmol) and 105 μL of FMA, (0.57 mmol)). After 3 freeze–pump–thaw cycles, the reaction mixture was irradiated with a blue LED (wavelength 435 nm). After 24 h, samples were dissolved in CDCl_3_ and conversions of monomers were calculated according to NMR spectroscopy data. In order to achieve purification, polymers were reprecipitated from chloroform to methanol. Polymers were separated from the solution by centrifugation and dried in vacuum.

## 3. Results and Discussion

### 3.1. Polymerization of FMA

**Figure 1:**
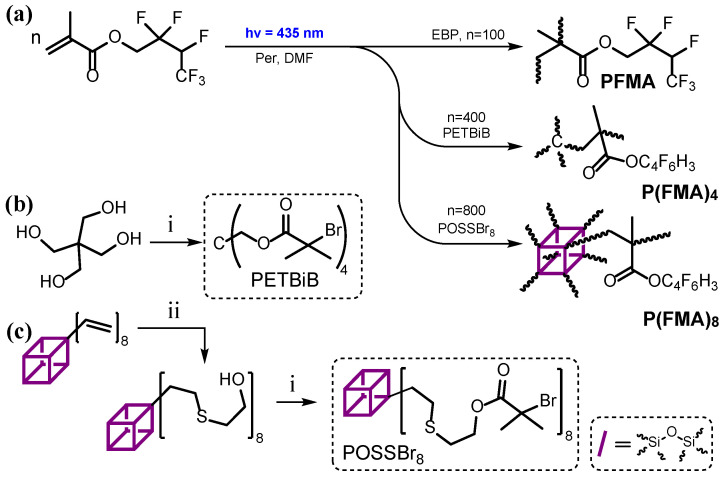
Synthesis of fluorinated polymers **PFMA**, **P(FMA)_4_** and **P(FMA)_8_** (**a**) and tetrafunctional (PETBiB) (**b**) and octafunctional (POSSBr_8_) initiators (**c**). Conditions: i—excess of 2-bromoisobutyryl bromide, TEA, THF 0–40 °C; ii—excess of 2-mercaptoethanol, DBPO, THF, UV irradiation.

In the first step of our research, we synthesized a series of 2,2,3,4,4,4-hexafluorobutyl methacrylate (**FMA**)-based polymers of different architectures (linear and star-shaped) (Figure 1). The organocatalytic atom transfer radical photopolymerization (O-ATRP) of **FMA** was conducted using perylene (Per) as a photocatalyst in conjunction with corresponding alkyl bromides as initiators in DMF under blue light irradiation (Figure S2). Perylene was selected as a photocatalyst due to its high efficiency in O-ATRP of methacrylate-type monomers [58]. Ethyl 2-bromo-2-phenylacetate (EBP) was used as an initiator for the preparation of linear polymers, while PETBiB and POSSBr_8_ were utilized as tetrafunctional and octafunctional ATRP initiators for the synthesis of star-shaped architectures (**P(FMA)_4_**, **P(FMA)_8_**), respectively (Figure 1).

**Figure 2:**
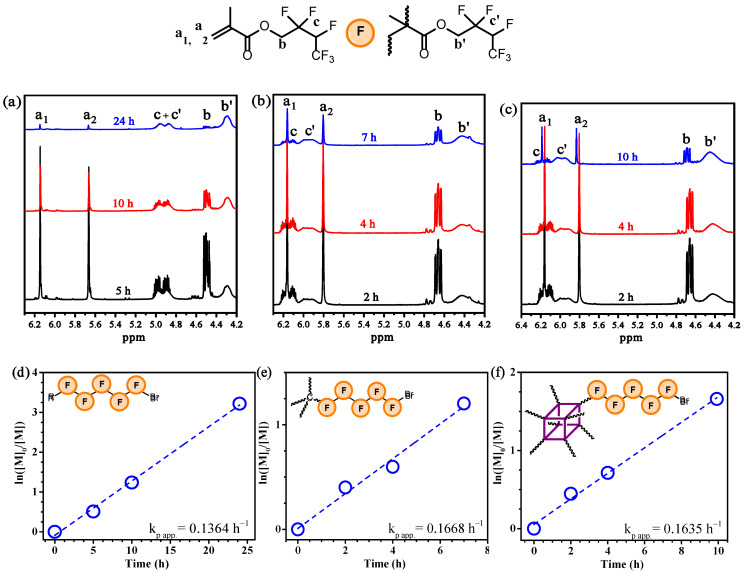
Fragments of ^1^H NMR spectra of polymerization mixtures of **PFMA** (CDCl_3_) (**a**), **P(FMA)_4_** (DMSO-d^6^) (**b**) and **P(FMA)_8_** (DMSO-d^6^) (**c**). First-order plots for polymerization of **FMA** using EPB (**d**), PETBiB (**e**) and POSSBr_8_ (**f**) as initiators. Polymerization conditions: [M]_0_/[I]_0_ = 100, 400 and 800 for **PFMA**, **P(FMA)_4_** and **P(FMA)_8_**, respectively; [M]_0_/[Per]_0_ = 900/1, [M]_0_ = 2.7 M, 435 nm, r.t., in DMF.

In order to investigate the kinetics of the process, samples of the reaction mixture were taken at a predetermined time and then were examined using ^1^H NMR spectroscopy (Figure 2). Monomer conversions were calculated using proton signals of the monomer’s and polymer’s fluoroalkyl group (**b**, **c**, **b’**, **c’**) as well as the signals of methylene protons of the methacrylate group (**a**) (Figure 2a–c) according to the following equations:

(1) $$Conv.=\frac{I\left(c+c^{\prime }\right)-I(a_{1})}{I\left(c+c^{\prime }\right)}\;or\;\frac{I(b\prime )}{I\left(b\right)+I(b\prime )}\;for\;PFMA$$

(2) $$Conv.=\frac{I\left(a_{1}+c+c^{\prime }\right)-2I(a_{2})}{I\left(a_{1}+c+c^{\prime }\right)-I(a_{2})}\;or\;\frac{I(b\prime )}{I\left(b\right)+I(b\prime )}\;for\;4p\left(FMA\right),\;8p(FMA),$$

where I(**x**)—the integral intensity of the corresponding signal. Both Equations (1) and (2) demonstrated convergence with each other (ΔConv. < 2%), and for further kinetic studies the average values between two calculated conversions were used.

As shown in Figure 2d–f, the first-order plots were found to be linear, indicating that concentration of active species remains constant during polymerizations. The rate of **FMA** polymerization does not depend on the nature of the initiator due to the similar structures of the initiators and the same concentration of initiating centers: k_p app._ = 0.14 h^−1^, k_p app._ = 0.17 h^−1^ and k_p app._ = 0.16 h^−1^ for **PFMA**, **P(FMA)_4_** and **P(FMA)_8_**, respectively.

Despite the linearity of the first-order plots, molar masses of synthesized polymers almost did not change with the monomer conversion, and dispersity was rather high (Table 1), while SEC curves did not change with conversion (Figure S3a), indicating the operation of side reactions. Nevertheless, the M_n_(SEC) values at complete conversion increased with the increase in the number of arms for star-shaped polymers as compared to their linear counterpart, which was attributed to the higher initial [M]_0_/[I]_0_ ratio (100 for **PFMA**, 400 for **P(FMA)_4_** and 800 for **P(FMA)_8_**) (Figure S3). It should be noted that for star-shaped polymers the molar masses are underestimated since they were measured by conventional SEC against polystyrene standards. Therefore, these values could be used only for the qualitative explanation of the observed trends.

**Figure 3:**
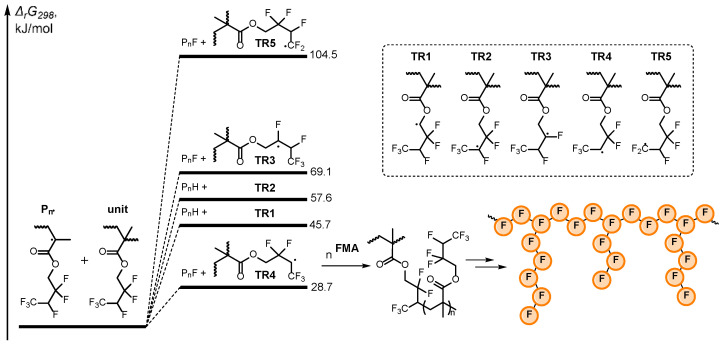
Potential chain transfer processes and their calculated Gibbs free energies.

We hypothesized that the above-mentioned side reaction could be a chain transfer to the polymer involving the fluoroalkyl fragments of the monomer unit. In order to estimate the probability of such a process, we carried out corresponding computations using the B3LYP/6-31G(d) theory level with a generic CPCM solvation model to account for DMF influence. The chain transfer process was considered as a reaction of the growing radical (P_n_) with the monomer unit, leading to the formation of five possible transfer radicals **TR1-5** (Figure 3). It should be noted that **TR1,2** radicals are generated due to the hydrogen atom transfer, while the others (**TR3-5**) are formed in the course of the fluorine atom transfer. In order to estimate the intensity of the chain transfer processes, comparison of thermodynamic stabilities of the resulting radicals was performed. The chosen approach is reliable, as indicated by its consistency with the Bell–Evans–Polanyi principle stating that thermodynamic and kinetic parameters of analogous processes correlate with each other in a similar way [59,60].

According to the computed data, all of the considered chain transfer processes are thermodynamically unfavorable (Figure 3, Table S1). Moreover, the reactions, leading to the formation of radicals localized on a carbon atom bonded with fluorine, are especially endothermic (**TR2,3,5**, Figure 3). This observation is consistent with known experimental data indicating that a fluorine atom as a substituent in the α-position destabilizes radicals because it turns their hybridization close to sp^3^ (pyramidal structure), while the F atom, which is located in the β-position, stabilizes spin-density by hyperconjugation even slightly better than alkyl groups [61,62]. Thus, after theoretical analysis, it can be concluded that the most probable chain transfer process is the one leading to the formation of **TR4**. Despite the reaction being fairly endothermic, this process is possible and could be responsible for the observed increase in dispersity since it leads to the formation of additional active growth centers on the chain, and consequently, a *graft*-polymer fraction is produced.

Another possible side reaction leading to an increase in dispersity could be competitive self-polymerization of **FMA** under visible light irradiation, which was reported for different types of methacrylate monomers [63,64,65,66]. In order to evaluate the contribution of this side reaction in our conditions, the polymerization of **FMA** without addition of the initiator was performed. A monomer conversion of 8% was obtained in 24 h (Figure S4), indicating the insignificant contribution of self-polymerization, which is often strongly inhibited in the presence of ATRP initiators [64,65,66,67].

**Figure 4:**
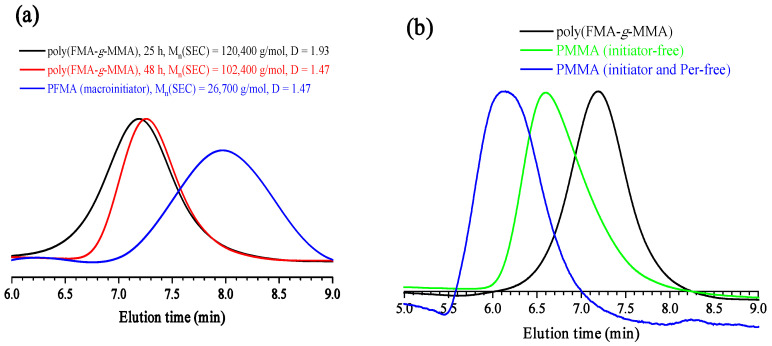
SEC curves of the graft-copolymer with an **FMA**-based main chain and MMA-based side chains and a macroinitiator (**PFMA**) utilized for its synthesis (**a**). Comparison of the SEC curves of different MMA-based (co)polymers, which were synthesized under 25 h of blue light irradiation in the following various conditions: presence or absence of Per or **PFMA** as a photocatalyst and a macroinitiator, respectively (**b**).

Finally, fluorinated compounds can act as initiators in ATRP processes [68,69,70]; therefore, the formation of *graft*-copolymers via activation of carbon–fluorine bonds by the excited state of Per in both monomers and polymers could also be the reason for the loss of control during O-ATRP of **FMA**. To prove this, **PFMA** was synthesized by conventional AIBN-initiated radical polymerization of FMA (Figure 4a and Figure S5, Appendix A.1, Table S2, Entry 1) and used as a macroinitiator for O-ATRP of methyl methacrylate (MMA) in the same conditions as for O-ATRP of **FMA** (Table S2, Entry 2).

It was found that polymerization of MMA proceeds smoothly, affording the polymer with 61% conversion after 25 h of irradiation (Figure S6). The molar mass of the polymer increased significantly, while SEC traces shifted to the high-molecular-weight region, indicating efficient grafting of MMA to the **PFMA** backbone (Figure 4a). The dispersity of the synthesized polymer became lower with increasing reaction time, but the number-average molecular weight did not change significantly, which may be consistent with underestimation of M_n_ by conventional SEC due to the formation of a graft-copolymer (Figure 4a).

Several control experiments were then performed to estimate the contribution of self-polymerization of MMA. First, photoinduced polymerization of MMA without the addition of an initiator proceeded at a lower rate, affording PMMA with a much higher molar mass and dispersity as compared to polymerization with **PFMA** as a macroinitiator (Figure 4b and Figure S7, Table S2, Entry 3). Moreover, slow self-polymerization of MMA was able to proceed even without Per producing polymers with very high molar masses (Figure 4b, Table S2, Entry 4). Therefore, based on the results of control experiments, we can conclude that the contribution of self-polymerization of MMA is not significant.

Taking into account the obtained results, two side reactions could operate during the O-ATRP of **FMA**: (i) chain transfer to the polymer or monomer via fluorine atom abstraction (see Figure 3 and the discussion therein) and (ii) the activation of carbon–fluorine bonds by the excited state of the photocatalyst (see Figure 4 and the discussion therein). Both of these side reactions result in the formation of branched and/or *graft*-copolymers, which is confirmed by the analysis of SEC traces (Figure 3). These side reactions resulted in broadening of molecular weight distribution and the appearance of an insignificant high-molecular-weight fraction in the case of the preparation of the linear **PFMA** polymer (Figure S3a). A more dramatic influence of side reactions was observed in the synthesis of star-shaped polymers with four arms, leading to the appearance of a series of peaks in the high-molecular-weight region (Figure S3b), which indicates the formation of coupled stars. The star–star coupling is less pronounced in the case of the preparation of **P(FMA)_8_**_,_ probably due to the more compact structure and shorter arms (Table 1).

### 3.2. Polymerization of IBSS

**Figure 5:**
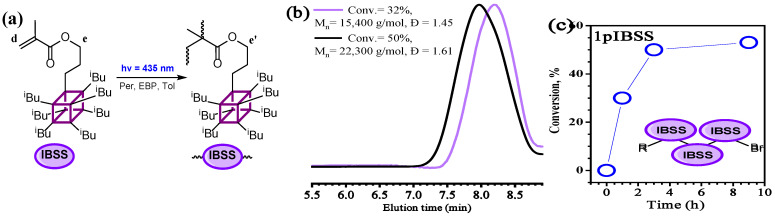
Polymerization scheme of **IBSS** (**a**), with corresponding SEC traces (**b**) and kinetic plot (**c**). Polymerization conditions: [M]_0_ = 1 g/mL (1.06 M), [M]_0_/[I]_0_/[Per]_0_ = 900/9/1, 435 nm, r.t., toluene.

In the next step of this study, the photoinduced O-ATRP of a polyhedral oligomeric silsesquioxane (POSS)-based methacrylate-type monomer (**IBSS**) was tested using the same photoinitiating system (Figure 5a). It is known that the polymerization of IBSS is challenging, primarily due to its low ceiling temperature (T_c_), which is caused by the steric hindrances created by the bulk side group [71]. Therefore, O-ATRP was hypothesized to be a more efficient approach, as it allows radical polymerization to proceed at room temperature.

Indeed, O-ATRP of **IBSS** with EBP as an initiator and Per as a photocatalyst resulted in linear polymers (**PIBSS**) with M_n_ up to 70,800 g mol^−1^ and relatively low dispersities (Đ = 1.45–1.61, Figure 5b, Table 2). Although the monomer conversion leveled off around 50% (Figure 5c), the number-average molar mass of synthesized polymers increased with increasing conversion, while SEC curves completely shifted to the high-molecular-weight region (Figure 5b).

Previous research data make it possible to calculate absolute M_n_ values for the analyzed polymer using the following equation [72]:

(3) $$\begin{array}{c} M_{n}\left(Corr.\right)=3.6906\times M_{n}\left(SEC\right)-12982\;g/mol,\\ M_{n}(SEC)∊(12000;29000)\;g/mol \end{array}$$

Accepting the calculated values (Table 2, M_n_(Corr.)) as absolute makes it possible to estimate the initiation efficiency (IE) for the studied polymerization process. The calculations carried out for samples obtained at different conversions show close agreement of the obtained IE values—68.9% for 1 h, 68% for 9 h and 71% for 24 h—indicating the relative reliability of the proposed method for estimating IE. It should be noted that the observed IE is fairly high in comparison with that for MMA polymerization in similar conditions (40% in benzene and 9% for DMF medium [58]).

It should be noted that attempting the preparation of a star-shaped polymer using PETBiB as a tetrafunctional initiator results in very low monomer conversion (7%), affording the polymer with a relatively low molecular weight (Table 2, Figure S8). Since the activity of all initiators was similar in O-ATRP of FMA (vide supra) and ceiling temperature would not depend on the initiator’s nature, the steric hindrance is most probably responsible for the observed low monomer conversion obtained during **IBSS** polymerization with PETBiB as an initiator.

**IBSS** conversion to a polymer for both experiments was calculated using ^1^H NMR spectroscopy according to the following formula (Figure S9):

(4) $$Conv.=\frac{I\left(e+e^{\prime }\right)-I(d)}{I\left(e+e^{\prime }\right)}\;or\;\frac{I(e\prime )}{I\left(e\right)+I(e\prime )}$$

### 3.3. Copolymerization of FMA with IBSS

**Figure 6:**
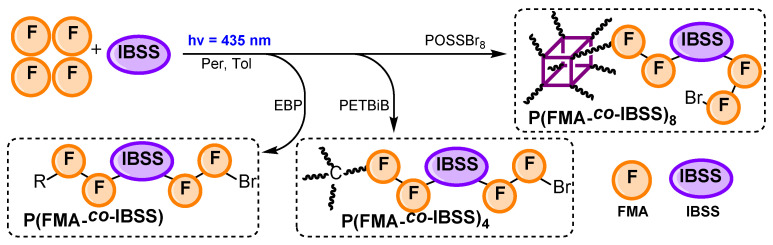
Copolymerization of **FMA** with **IBSS** using different initiators. Copolymerization conditions: [M]_0_/[I]_0_ = 100, 400 and 800 for **P(FMA-*co*-IBSS)**, **P(FMA-*co*-IBSS)_4_** and **P(FMA-*co*-IBSS)_8_**, respectively; [FMA]_0_/[IBSS]_0_/[Per]_0_ = 720/180/1, [FMA]_0_ + [IBSS]_0_ = 3.4 M, 435 nm, r.t., in toluene.

Further, hybrid copolymers containing both fluorinated moieties and POSS fragments with different architectures (**P(FMA-*co*-IBSS)**, **P(FMA-*co*-IBSS)_4_** and **P(FMA-*co*-IBSS)_8_**) were prepared via random O-ATRP using mono-, tetra- and octafunctional initiators, respectively (Figure 6).

**Figure 7:**
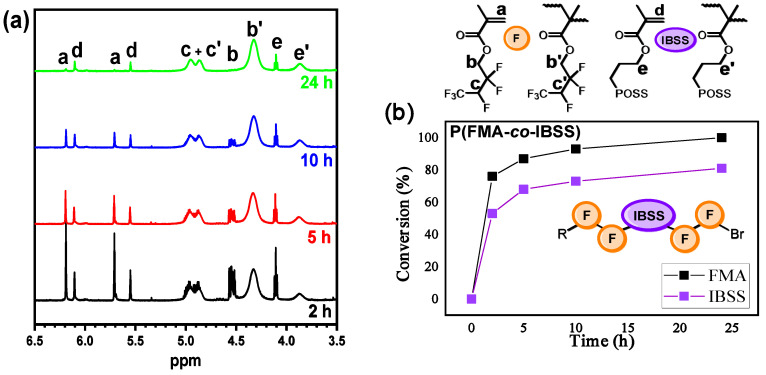
^1^H NMR spectra of **P(FMA-*co*-IBSS)** polymerization mixture, recorded in CDCl_3_ (**a**). Kinetic plot of **FMA** and **IBSS** copolymerization using EBP (**b**). Copolymerization conditions: [FMA]_0_ + [IBSS]_0_ = 3.4 M, [FMA]_0_/[IBSS]_0_/[I]_0_/[pe]_0_ = 720/180/9/1, 435 nm, r.t., in toluene.

The kinetics of the copolymerization of **FMA** and **IBSS** with a monofunctional initiator was first studied by looking at the consumption of both monomers by ^1^H NMR spectroscopy (see Equations (1) and (4) for the calculation of the conversion) (Figure 7, Table 3).

The obtained data show that **FMA** exhibits higher activity than **IBSS** in the studied copolymerization processes. This is likely due to its smaller side group, which causes less steric hindrance compared to the bulky POSS fragment. Nevertheless, **IBSS** conversion in copolymerization reached much higher values than in its homopolymerization (up to 81%). This indicates that the rate constant for IBSS adding to an IBSS-ended chain (k_IBSS/IBSS_) is lower than for addition to an **FMA**-ended chain (k_IBSS/FMA_), giving a reactivity ratio of r_IBSS_ = k_IBSS/IBSS_/k_IBSS/FMA_ < 1 according to the Mayo–Lewis equation [19,20]. This also explains the sharp slowdown of IBSS copolymerization at reduced **FMA** concentrations: more **IBSS**-ended chains form, and their low k_IBSS/IBSS_ value hinders further IBSS incorporation [73,74].

The copolymerization of **FMA** with **IBSS** proceeded up to complete monomer conversion in contrast to homopolymerization of IBSS, affording random copolymers with a relatively high molecular weight and moderate dispersity (Table 3). After successful preparation of linear copolymers, the synthesis of star-shaped copolymers was then targeted. The four- and eight-arm copolymers were synthesized via copolymerization of **FMA** and **IBSS** using PETBiB and POSSBr_8_ as initiators and Per as a photocatalyst. Although high-molecular-weight (M_n_ > 80,000 g mol^−1^) star-shaped copolymers were synthesized with a high monomer conversion, these copolymers are characterized by much higher dispersity as compared to their linear counterparts (Table 3). It should be noted that, similarly to the synthesis of star-shaped homopolymers (**P(FMA)_4_**), the SEC trace of **P(FMA-*co*-IBSS)_4_** is multimodal, indicating significant star–star coupling (Figure S10). For all resulting copolymers, the quantity units in the polymer chain χ(FMA) were in the 81–86% range (χ(IBSS) = 14–19%), which is quite close to the composition of the initial monomer mixture (χ_0_(FMA) = 80%, χ_0_(IBSS) = 20%).

### 3.4. Thermal Properties

Thermal properties of the synthesized polymers were studied using DSC and TGA analyses. The obtained data are summarized in Table 4 and Figures S11 and S12. Evidently, the thermal stability of the materials under investigation differed depending on the polymer composition and architecture. Thus, among the linear polymers studied, the T_ID_ decreased in the following series: **PIBSS** > **PFMA** > **P(FMA-*co*-IBSS)**. At the same time, there is a clear trend towards an increase in T_ID_ with increasing complexity of the architecture for **FMA**-based homopolymers (from 256 to 306 °C) and random copolymers (from 239 to 292 °C).

In the series of linear polymers, an increase in the proportion of **IBSS** was observed to result in an increase in glass transition temperature (T_g_) from 53 to 85 °C. A similar trend was observed for four- and eight-armed star-shaped polymers (Table 4). It is noteworthy that the transition from linear to star-shaped polymers resulted in an increase in T_g_. As is known from the literature, glass transition temperatures of star-shaped polymers are commonly lower than those of their linear counterparts because of the higher mobility of polymer chains in star-shaped polymers due to greater free volume [75,76]. Therefore, the observed increase in T_g_ is likely associated with an increase in the molecular weight of the materials under investigation.

### 3.5. Solvophobic Properties

The measurement of the contact angle is a reliable technique for the characterization of solid surfaces and the determination of their wettability and surface tension. In order to evaluate the hydrophobicity and oleophobicity of the obtained materials, a drop of liquid (distilled water or vegetable oil) was applied to glasses with a thin film of the corresponding polymer. The obtained data are presented in Table 5 and Figures S13 and S14. For comparative purposes, the contact angles were also measured on a glass surface, yielding values of 61.2° for water and 45.9° for oil, establishing a baseline.

The data from Table 5 reveal that the polymer films exhibit significantly higher water contact angles than the glass baseline. The homopolymers **PFMA**, **P(FMA)_4_**, **P(FMA)_8_** and **PIBSS**, along with the copolymers **P(FMA-*co*-IBSS)**, **P(FMA-*co*-IBSS)_4_** and **P(FMA-*co*-IBSS)_8_**, showed angles ranging from approximately 90° to 97.5°. Among them, linear **PFMA** and **PIBSS** demonstrated the highest hydrophobicity. A study of a series of homopolymers based on **FMA** reveals that hydrophobicity exhibits a negligible decrease with increasing polymer architecture complexity. Conversely, random copolymers demonstrate an opposing trend due to the effect of POSS presence in their structures.

In contrast to the water measurements, the oil contact angles for all polymer films were low and close to the value for pure glass (45.9°). Contact angles of the studied polymer films with vegetable oil were found to be ~46° for **PFMA**, **P(FMA)_4_** and **P(FMA)_8_**, ~33° for **PIBSS**, and ~41° for random copolymers **P(FMA-*co*-IBSS)**, **P(FMA-*co*-IBSS)_4_** and **P(FMA-*co*-IBSS)_8_**. **PIBSS** exhibited the smallest contact angle, which indicates it has the highest oleophilicity in comparison with the other polymers examined.

It is generally considered that the contact angle for solvophilic surfaces is less than 90°, while for solvophobic ones it is θ > 90° [23]. Consequently, all of the studied polymer film surfaces exhibit hydrophobic and oliophilic properties.

### 3.6. Atomic Force Microscopy

**Figure 8:**
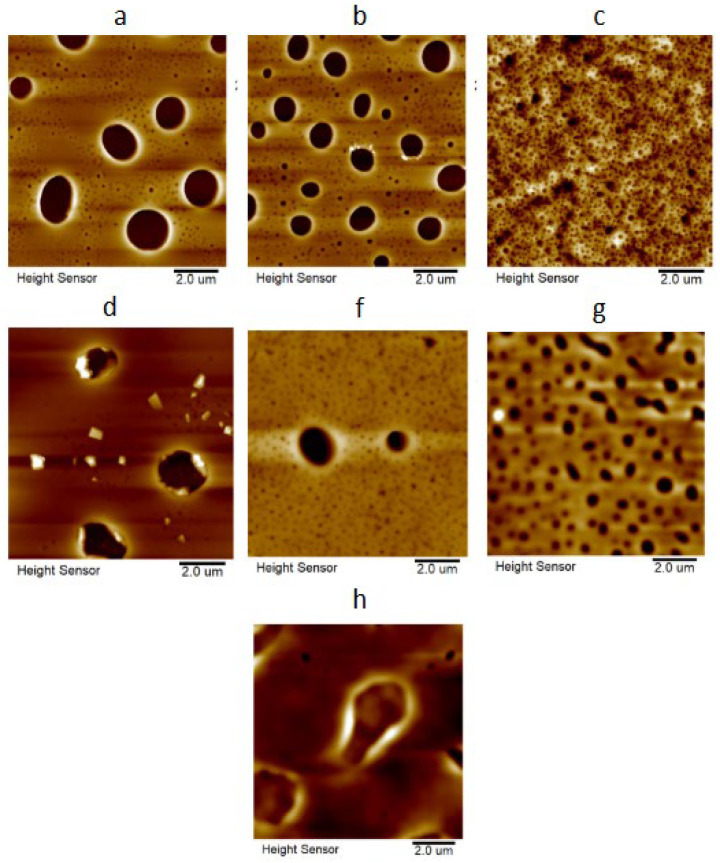
AFM image of the surface topography of the obtained films: **PFMA** (**a**), **P(FMA)_4_** (**b**), P(FMA)_8_ (**c**), **P(FMA-co-IBSS)** (**d**), **P(FMA-co-IBSS)_4_** (**f**), **P(FMA-co-IBSS)_8_** (**g**) and **PIBSS** (**h**).

The obtained polymer films were further investigated by atomic force microscopy. They exhibited inhomogeneity, with pores of varying sizes being clearly visible on their surface (Figure 8), which was expected as it is well-known that fluorinated polymers are widely used for the preparation of porous membranes for different applications [15,23,25]. This phenomenon is mainly attributed to the fact that fluorinated chains have minimal interactions with each other, thus leaving voids after solvent evaporation. It has been observed that pore sizes decrease with the increase in polymer arms for both homopolymers and copolymers, which demonstrates that fluorinated segments in linear (co)polymers much more easily spatially separate from each other than those in sterically hindered star-shaped (co)polymers.

The surface roughness of thin films was measured in air. To obtain more reliable values, surface roughness was calculated over the entire image area as well as over selected areas that excluded large holes. Obtained thickness and surface roughness values are collected in Table 6.

It was observed that the increase in the branching of the **FMA** homopolymer results in a decrease in the surface roughness of the corresponding films, which is clearly the cause of the earlier observed decrease in hydrophobicity for the **PFMA**, **P(FMA)_4_** and **P(FMA)_8_** series [77,78]. In the case of the obtained copolymer films, no direct correlation between roughness and branching of macromolecules was found. Consequently, the earlier observed increase in θ values in the series **P(FMA-*co*-IBSS)**, **P(FMA-*co*-IBSS)_4_** and **P(FMA-*co*-IBSS)_8_** can be attributed to the classical effect of increasing hydrophobicity with increasing branching of the polymer containing **IBSS** monomeric units, which are more hydrophobic than **FMA** [24].

## 4. Conclusions

In this work, the potential of using of O-ATRP for the (co)polymerization of fluorinated (**FMA**) and POSS-containing (**IBSS**) methacrylates was explored for the first time to successfully synthesize linear and four-armed and eight-armed star-shaped (co)polymers. However, the chain transfer to the polymer via either fluoride abstraction by the growing macroradical or activation of carbon–fluorine bonds by the excited state of the photocatalyst was operated under the investigated conditions, resulting in formation of branched polymer chains and coupled stars in the synthesis of linear and star-shaped (co)polymers, respectively. Nevertheless, it was demonstrated that the morphology, thermal stability and hydrophobicity of films prepared from synthesized (co)polymers could be controlled by the copolymer composition and architecture. The obtained hybrid films demonstrated high thermal stability (up to 306 °C), roughness (up to 34 nm) and hydrophobicity (up to 98°). The obtained polymers hold particular promise for membrane technologies, where they have the potential to enhance chemical resistance, reduce fouling and ensure long-term operational stability in aggressive environments. In addition, based on both their high thermal/chemical stability and their hydrophobicity, POSS-containing copolymers are of interest as special coatings.

## Figures and Tables

**Table 1:** Photoinduced O-ATRP of **FMA** with different initiators, using perylene as photocatalyst in DMF ^1^.

Polymer	Time (h)	Conv. ^2^ (%)	Mn(SEC) (g/mol)	Đ
**PFMA**	5	40	26,600	1.77
	10	71	22,700	2.05
	24	96	25,600	1.73
	48	100	28,100	1.41
**P(FMA)_4_**	2	33	46,000	1.37
	4	45	40,700	1.52
	7	70	38,000	1.45
	24	100	37,400	1.59
**P(FMA)_8_**	2	36	53,100	1.78
	4	51	47,700	1.41
	10	81	42,800	1.89
	48	100	38,100	2.08

^1^ Polymerization conditions: [M]_0_/[I]_0_ = 100, 400 and 800 for **PFMA**, **P(FMA)_4_** and **P(FMA)_8_**, respectively; [M]_0_/[Per]_0_ = 900/1, [M]_0_ = 2.7 M, 435 nm, r.t., in DMF. ^2^ Determined by ^1^H NMR spectroscopy.

**Table 2:** Photoinduced O-ATRP of **IBSS** with different initiators using perylene as photocatalyst in toluene ^1^.

Polymer	Time (h)	Conv. ^2^ (%)	M_n_(Theor.) (g/mol)	M_n_(SEC) (g/mol)	M_n_(Corr.) ^3^ (g/mol)	IE ^4^ (%)	Đ
**PIBSS**	1	32	30,200	15,400	43,800	68.9	1.45
	9	50	47,200	22,300	69,300	68.1	1.61
	24	53	50,000	22,700	70,800	70.6	1.60
**P(IBSS)_4_**	24	7	26,400	2200	-	-	1.13

^1^ Polymerization conditions: [M]_0_/[I]_0_ = 100 and 400 for **PIBSS** and **P(IBSS)_4_**, respectively, [M]_0_/[Per]_0_ = 900/1, [M]_0_ = 1 g/mL (1.06 M), 435 nm, r.t., toluene. ^2^ Determined by ^1^H NMR spectroscopy. ^3^ The number-average molar masses calculated using the correlation Equation (3) [72]. ^4^ Calculated as M_n_(theor.)/M_n_(Corr.).

**Table 3:** Photoinduced copolymerization of FMA with IBSS with different initiators using perylene as photocatalyst in toluene ^1^.

Polymer	Time (h)	Conv. ^2^ (%)			χ(FMA)/χ(IBSS) ^3^	M_n_(SEC) (g/mol)	Đ
		FMA	IBSS	Common			
**P(FMA-*co*-IBSS)**	2	76	53	71	85/15	27,900	1.56
	5	87	68	83	84/16	27,300	1.88
	10	93	73	89	84/16	29,900	1.65
	24	100	81	96	83/17	30,100	1.64
**P(FMA-*co*-IBSS)_4_**	24	98	90	96	81/19	79,800	3.80
**P(FMA-*co*-IBSS)_8_**	24	92	62	86	86/14	87,300	2.74

^1^ [M]_0_/[I]_0_ = 100, 400 and 800 for **P(FMA-*co*-IBSS)**, **P(FMA-*co*-IBSS)_4_** and **P(FMA-*co*-IBSS)_8_**, respectively; [FMA]_0_/[IBSS]_0_/[Per]_0_ = 720/180/1, [FMA]_0_ + [IBSS]_0_ = 3.4 M, 435 nm, r.t., in toluene. ^2^ Conversions of **FMA** or **IBSS** into copolymer, determined by ^1^H NMR spectroscopy. ^3^ Copolymer composition.

**Table 4:** Thermal properties of synthesized (co)polymers.

Polymer	M_n_(SEC) (g/mol)	T_g_ ^1^ (°C)	T_ID_ ^2^ (°C)
**PFMA**	28,100	53	256
**P(FMA)_4_**	37,400	55	262
**P(FMA)_8_**	38,100	58	306
**P(FMA-*co*-IBSS)**	30,100	55	239
**P(FMA-*co*-IBSS)_4_**	79,800	67	259
**P(FMA-*co*-IBSS)_8_**	87,300	65	292
**PIBSS**	22,700	85	362

^1^ Determined by DSC from the second heating scan: scan rate 20 °C min^−1^. ^2^ The 5% weight loss was determined by TGA: heating rate 20 °C min^−1^.

**Table 5:** Contact angle values (θ) for the synthesized polymers.

Contact Liquid	Glass	PFMA	P(FMA)_4_	P(FMA)_8_	P(FMA-*co*-IBSS)	P(FMA-*co*-IBSS)_4_	P(FMA-*co*-IBSS)_8_	PIBSS
water	61.2 ± 3.1	96.1 ± 0.5	90.4 ± 0.4	89.2 ± 0.2	91.3 ± 2.1	93.6 ± 0.3	95.6 ± 0.2	97.5 ± 0.2
oil	45.9 ± 0.6	46.2 ± 2.3	46.5 ± 0.9	46.8 ± 1.9	41.8 ± 1.6	41.6 ± 2.0	42.1 ± 1.5	33.0 ± 1.2

**Table 6:** Thickness and surface roughness values of the studied polymer films.

Polymer	Film Thickness (nm)	Film Roughness (nm)	
		Whole Area	Without Counting Large Holes
**PFMA**	73.2 ± 13.2	33.7 ± 4.8	11.5 ± 0.9
**P(FMA)_4_**	102.4 ± 9.7	21.3 ± 3.1	7.5 ± 1.3
**P(FMA)_8_**	175.0 ± 9.2	6.7 ± 2.6	5.6 ± 2.5
**P(FMA-*co*-IBSS)**	105.9 ± 3.3	32.0 ± 15.8	11.6 ± 5.5
**P(FMA-*co*-IBSS)_4_**	190.9 ± 23.6	33.9 ± 12.4	13.3 ± 2.2
**P(FMA-*co*-IBSS)_8_**	114.4 ± 18.8	6.4 ± 1.0	6.4 ± 1.0
**PIBSS**	136.1 ± 1.8	18.3 ± 12.3	10.0 ± 2.6

## Data Availability

The original contributions presented in this study are included in the article/Supplementary Materials; further inquiries can be directed to the corresponding author.

## Supplementary Materials

The following supporting information can be downloaded at https://www.mdpi.com/article/10.3390/polym18010141/s1. Figure S1. ^1^H NMR spectra of PETBiB (top) and POSSBr_8_ in CDCl_3_ (bottom); Figure S2. The set-up used for photopolymerization (a); electroluminescence spectrum of applied diode (LED tape: 435 nm, 5.6 W) (b); Figure S3. SEC-curves for synthesized PFMA (a), P(FMA)_4_ (b) and P(FMA)_8_ (c); Figure S4. ^1^H NMR spectrum (DMSO-d^6^) of initiator-free FMA polymerization mixture after being irradiated for blue light for 24 h; Figure S5. ^1^H NMR spectrum (DMSO-d^6^) of PFMA reaction mixture (Entry 1, Table S2); Figure S6. ^1^H NMR spectrum (DMSO-d^6^) of MMA polymerization mixture after being irradiated for blue light for 25 h in presence of PFMA as C-F initiator (Entry 2, Table S2); Figure S7. ^1^H NMR spectrum (DMSO-d^6^) of initiator-free MMA polymerization mixture after being irradiated for blue light for 25 h (Entry 3, Table S2); Figure S8. SEC-curves for synthesized P(IBSS)_4_; Figure S9. ^1^H NMR spectrum (DMSO-d^6^) of IBSS polymerization mixture; Figure S10. SEC curves for the synthesized random copolymers; Figure S11. DSC curves of the synthesized (co)polymers; Figure S12. TGA curves of the synthesized (co)polymers; Figure S13. Measurement of contact angles with water (an image of one of the measurements is presented for each polymer film); Figure S14. Measurement of contact angles with vegetable oil (an image of one of the measurements is presented for each polymer film); Table S1. Thermodynamical parameters for hypothesized structures, calculated using B3LYP/6-31G(d) theory level with generic CPCM solvation model (DMF); Table S2. Investigation of carbon-fluorine bond activation.

## Abbreviations

The following abbreviations are used in this manuscript:

ATRP	Atom-transfer radical polymerization
DBPO	Diphenyl(2,4,6-trimethylbenzoyl)phosphine oxide
DFT	Density functional theory
**IBSS**	Methacryloxypropyl-substituted poly(isobutyl-T_8_-silsesquioxane)
**FMA**	2,2,3,4,4,4-Hexafluorobutyl methacrylate
MMA	Methyl methacrylate
O-ATRP	Organocatalyzed atom-transfer radical polymerization
Per	Perylene
**PIBSS**	Linear poly(methacryloxypropyl-substituted poly(isobutyl-T_8_-silsesquioxane))
**P(IBSS)_4_**	4-armed star-shaped poly(methacryloxypropyl-substituted poly(isobutyl-T_8_-silsesquioxane))
**PFMA**	Linear poly(2,2,3,4,4,4-hexafluorobutyl methacrylate)
**P(FMA)_4_**	4-armed star-shaped poly(2,2,3,4,4,4-hexafluorobutyl methacrylate)
**P(FMA)_8_**	8-armed star-shaped poly(2,2,3,4,4,4-hexafluorobutyl methacrylate)
**P(FMA-*co*-IBSS)**	Linear poly(2,2,3,4,4,4-hexafluorobutyl methacrylate-*co*-methacryloxypropyl-substituted poly(isobutyl-T_8_-silsesquioxane))
**P(FMA-*co*-IBSS)_4_**	4-armed star-shaped poly(2,2,3,4,4,4-hexafluorobutyl methacrylate-*co*-methacryloxypropyl-substituted poly(isobutyl-T_8_-silsesquioxane))
**P(FMA-*co*-IBSS)_8_**	8-armed star-shaped poly(2,2,3,4,4,4-hexafluorobutyl methacrylate-*co*-methacryloxypropyl-substituted poly(isobutyl-T_8_-silsesquioxane))
PMMA	Poly(methyl methacrylate)
THF	Tetrahydrofuran

## Appendix A

### Appendix A.1. Additional Polymerization Experiments

*AIBN-initiated radical polymerization of **FMA**.* In this polymerization experiment (Entry 1, Table S2), a solution of AIBN in DMF (0.25 mL, 6 mass.%) was added to FMA (250 μL, 1.35 mmol). After three freeze–pump–thaw cycles, polymerization was started by irradiation with a blue LED (wavelength 435 nm). After 28 h the polymer was separated from the reaction mixture by precipitation to water, and then dissolved in THF and reprecipitated to n-hexane (the procedure was performed twice). The resulting polymer (PFMA) was separated from the solution by centrifugation and dried in vacuum. Monomer conversion was determined gravimetrically.

*Synthesis of graft-copolymer with **FMA**-based core and MMA-based side chains.* In this polymerization experiment (Entry 2, Table S2), 1 mL of MMA, 2.6 mg of perylene, 23.5 mg of pFMA and 1 mL of DMF were added to a Schlenk tube. After three freeze–pump–thaw cycles, polymerization was started by irradiation with a blue LED (wavelength 435 nm). After a predetermined time, samples of the polymerization mixture were withdrawn and dissolved in CDCl3. Conversion of monomer was calculated using NMR spectroscopy data. In order to separate polymers from the reaction mixture, they were dissolved in THF and precipitated to methanol (the procedure was performed twice). Polymers were separated from the solution by centrifugation and dried in vacuum.

*Initiator-free perylene-catalyzed photopolymerization of MMA.* In this polymerization experiment (Entry 3, Table S2), 1 mL of MMA, 2.6 mg of perylene and 1 mL of DMF were added to a Schlenk tube. After three freeze–pump–thaw cycles, polymerization was started by irradiation with a blue LED (wavelength 435 nm). After a predetermined time, samples of the polymerization mixture were withdrawn and dissolved in CDCl3. Conversion of monomer was calculated using NMR spectroscopy data. In order to separate polymers from the reaction mixture, they were dissolved in THF and precipitated to methanol (the procedure was performed twice). Polymers were separated from the solution by centrifugation and dried in vacuum.

*Self-polymerization of MMA under blue light.* In this polymerization experiment (Entry 4, Table S2), 1 mL of MMA and 1 mL of DMF were added to a Schlenk tube. After three freeze–pump–thaw cycles, polymerization was started by irradiation with a blue LED (wavelength 435 nm). In order to separate polymers from the reaction mixture, they were dissolved in THF and precipitated to methanol (the procedure was performed twice). Polymers were separated from the solution by centrifugation and dried in vacuum.
