# Supplementary material for: Organocatalyzed Atom Transfer Radical (Co)Polymerization of Fluorinated and POSS-Containing Methacrylates: Synthesis and Properties of Linear and Star-Shaped (Co)Polymers
Source: Polymers (Basel). 2026 Jan 4;18(1):141. doi: 10.3390/polym18010141 (PMC12787686; doi:10.3390/polym18010141)
Supplement: Supplementary file 1 [file polymers-18-00141-s001.zip › polymers-4048568-supplementary.pdf]

Supplementary Materials for

Article

Organocatalyzed Atom Transfer Radical (Co)Polymerization of Fluorinated and POSS-Containing Methacrylates: Synthesis and Properties of Linear and Star-Shaped (Co)Polymers

Hleb Baravoi [1,2], Heorhi Belavusau [1,2,3], Aliaksei Vaitusionak [1,3], Valeriya Kukanova [3], Anastasia Frolova [3], Peter Timashev [3], Hongzhi Liu [4], and Sergei Kostjuk [5,*]

1 Research Institute for Physical Chemical Problems of the Belarusian State University, 14 Leningradskaya St., 220006 Minsk, Belarus; glebborovoj268@gmail.com (H.B.); george.vusau@gmail.com (H.B.); aliaksei.vaitusionak@gmail.com (A.V.)
2 Department of Chemistry, Belarusian State University, 14 Leningradskaya St., 220006 Minsk, Belarus
3 Institute for Regenerative Medicine, Sechenov First Moscow State Medical University, 8-2 Trubetskaya St., 119991 Moscow, Russia; kukanova_v_s@staff.sechenov.ru (V.K.); frolova_a_a_2@staff.sechenov.ru (A.F.); timashev_p_s@staff.sechenov.ru (P.T.)
4 School of Chemistry and Chemical Engineering, Shandong University, Jinan 250100, China; liuhongzhi@sdu.edu.cn
5 Equipe Chimie des Polymeres, Institut Parisien de Chimie Moleculaire, Sorbonne Universite, CNRS, 4 Place Jussieu, CEDEX 05, 75252 Paris, France
* Correspondence: sergei.kostjuk@sorbonne-universite.fr

Content

Figure S1. ^{1}H NMR spectra of PETBiB (top) and $POSSBr_8$ in $CDCl_3$ (bottom)........3

Figure S2. The set-up used for photopolymerization **(a)**; electroluminescence spectrum of applied diode (LED tape: 435 nm, 5.6 W) **(b)**........4

Figure S3. SEC-curves for synthesized **PFMA** (**a**), **P(FMA)$_4$** (**b**) and **P(FMA)$_8$** (**c**)........5

Table S1. Thermodynamical parameters for hypothesized structures, calculated using B3LYP/6-31G(d) theory level with generic CPCM solvation model (DMF)........6

Figure S4. ^{1}H NMR spectrum (DMSO-d^6) of initiator-free **FMA** polymerization mixture after being irradiated for blue light for 24 h........7

Figure S5. ^{1}H NMR spectrum (DMSO-d^6) of **PFMA** reaction mixture (Entry 1, Table S2)........7

Figure S6. ^{1}H NMR spectrum (DMSO-d^6) of MMA polymerization mixture after being irradiated for blue light for 25 h in presence of **PFMA** as C-F initiator (Entry 2, Table S2)........8

Figure S7. ^{1}H NMR spectrum (DMSO-d^6) of initiator-free **MMA** polymerization mixture after being irradiated for blue light for 25 h (Entry 3, Table S2)........9

Figure S8. SEC-curves for synthesized **P(IBSS)$_4$**........9

Figure S9. ^{1}H NMR spectrum (DMSO-d^6) of **IBSS** polymerization mixture........10

Figure S10. SEC curves for the synthesized random copolymers........10

Figure S11. DSC curves of the synthesized (co)polymers........11

Figure S12. TGA curves of the synthesized (co)polymers........11

Figure S13. Measurement of contact angles with water (an image of one of the measurements is presented for each polymer film)........12

Figure S14. Measurement of contact angles with vegetable oil (an image of one of the measurements is presented for each polymer film)........13

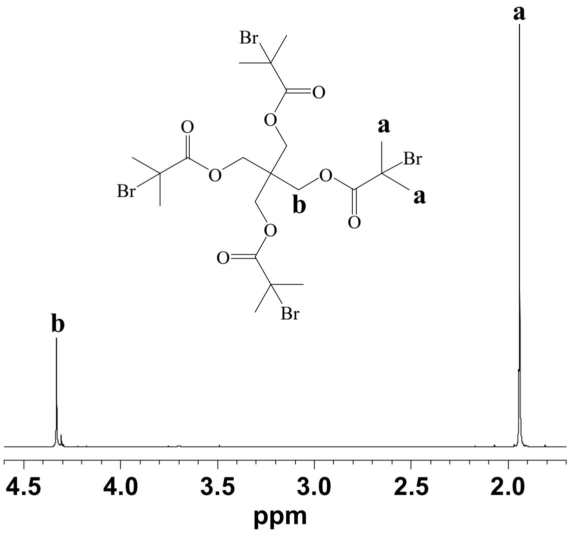

^{1}H NMR (500 MHz, $CDCl_3$) δ 4.33 (s, 2H), 1.94 (s, 6H).

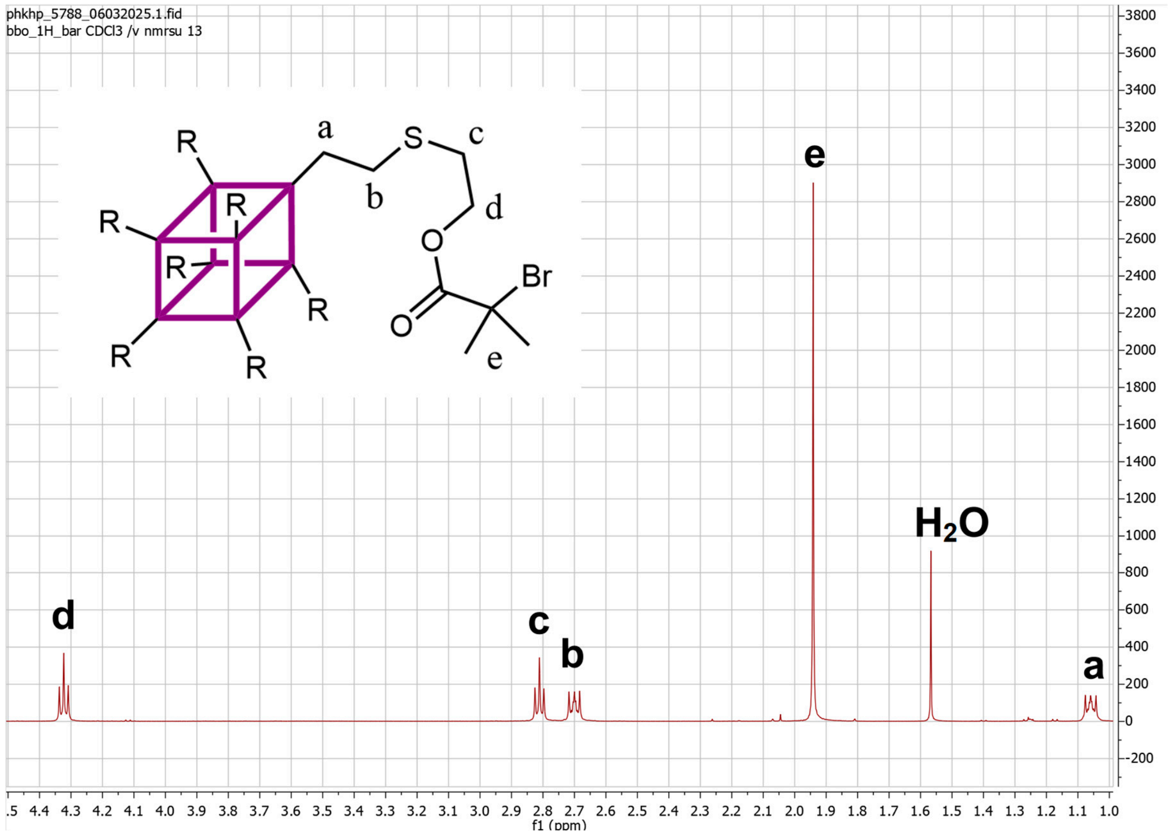

^{1}H NMR (500 MHz, $CDCl_3$) δ 4.32 (t, J = 7.1 Hz, 1H), 2.81 (t, J = 7.0 Hz, 1H), 2.74 – 2.67 (m, 1H), 1.94 (s, 3H), 1.09 – 1.02 (m, 1H).

Figure S1. ^{1}H NMR spectra of PETBiB (top) and $POSSBr_8$ in $CDCl_3$ (bottom).

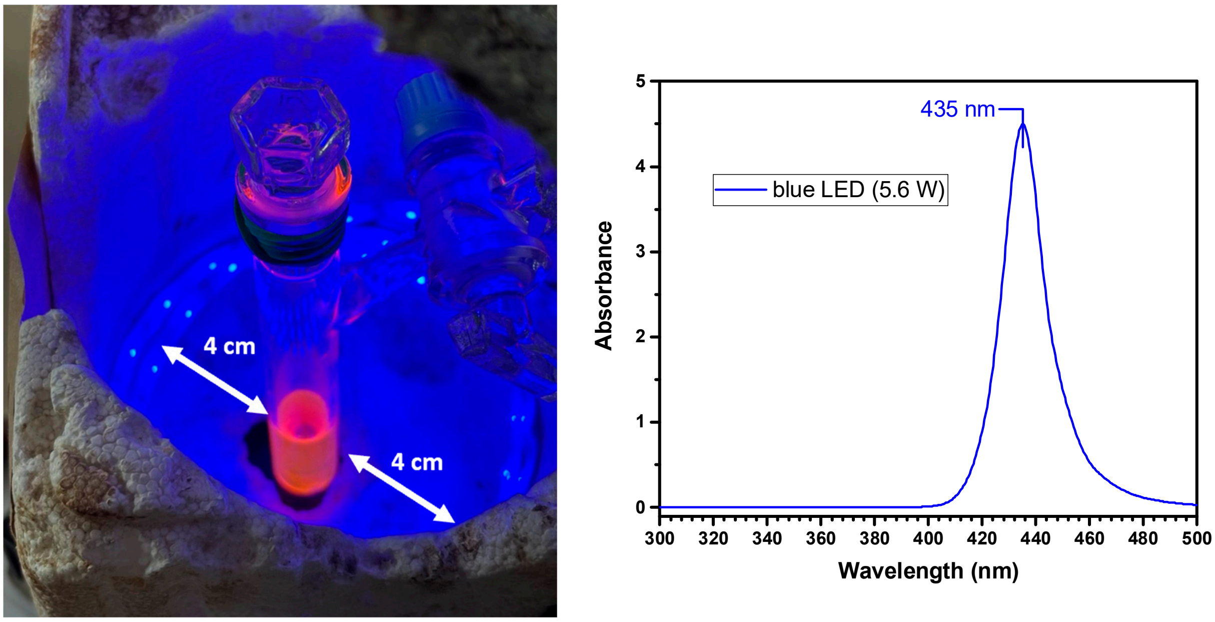

(a) **(b)**

Figure S2. The set-up used for photopolymerization **(a)**; electroluminescence spectrum of applied diode (LED tape: 435 nm, 5.6 W) **(b)**.

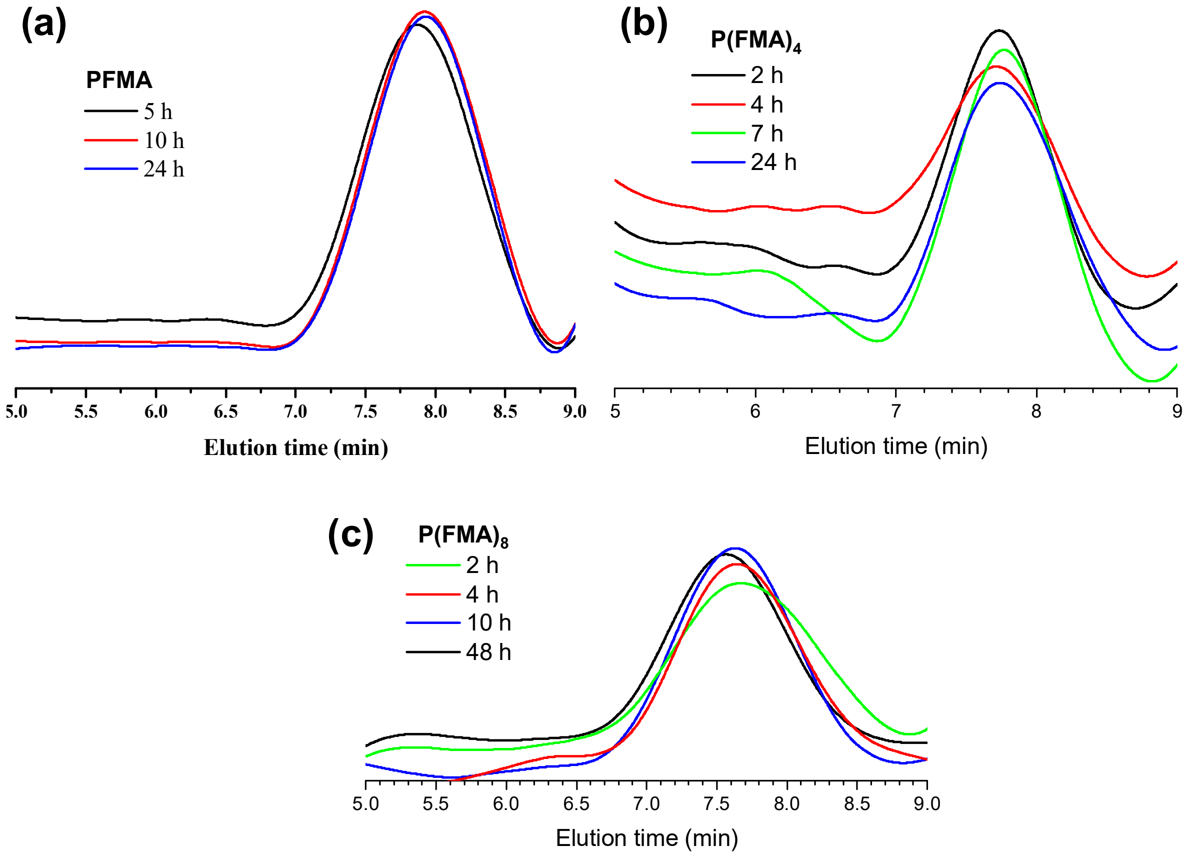

Figure S3. SEC-curves for synthesized **PFMA** (**a**), **P(FMA)$_4$** (**b**) and **P(FMA)$_8$** (**c**).

Table S1. Thermodynamical parameters for hypothesized structures, calculated using B3LYP/6-31G(d) theory level with generic CPCM solvation model (DMF)

Structure	**unit**	**$P_n\cdot$**	**P_nH**	**P_nF**	
G_{298}, Ha	-1060.159659	-1059.531852	-1060.159659	-1159.400343	
Structure	**TR1**	**TR2**	**TR3**	**TR4**	**TR5**
G_{298}, Ha	-1059.514465	-1059.50992	-960.264866	-960.280233	-960.251381

$$\textbf{unit} + \mathbf{P_n\cdot} \rightarrow \textbf{TR}(\mathbf{1} \text{ or } \mathbf{2}) + \mathbf{P_nH}$$

$\Delta_rG_{298} = G_{298}(\mathbf{TR}) + G_{298}(\mathbf{P_nH}) - G_{298}(\mathbf{P_n\cdot}) - G_{298}(\mathbf{unit})$ for **TR1** and **TR2**

$$\textbf{unit} + \mathbf{P_n\cdot} \rightarrow \textbf{TR(3-5)} + \mathbf{P_nF}$$

$\Delta_rG_{298} = G_{298}(\mathbf{TR}) + G_{298}(\mathbf{P_nF}) - G_{298}(\mathbf{P_n\cdot}) - G_{298}(\mathbf{unit})$ for **TR3**, **TR4** and **TR5**

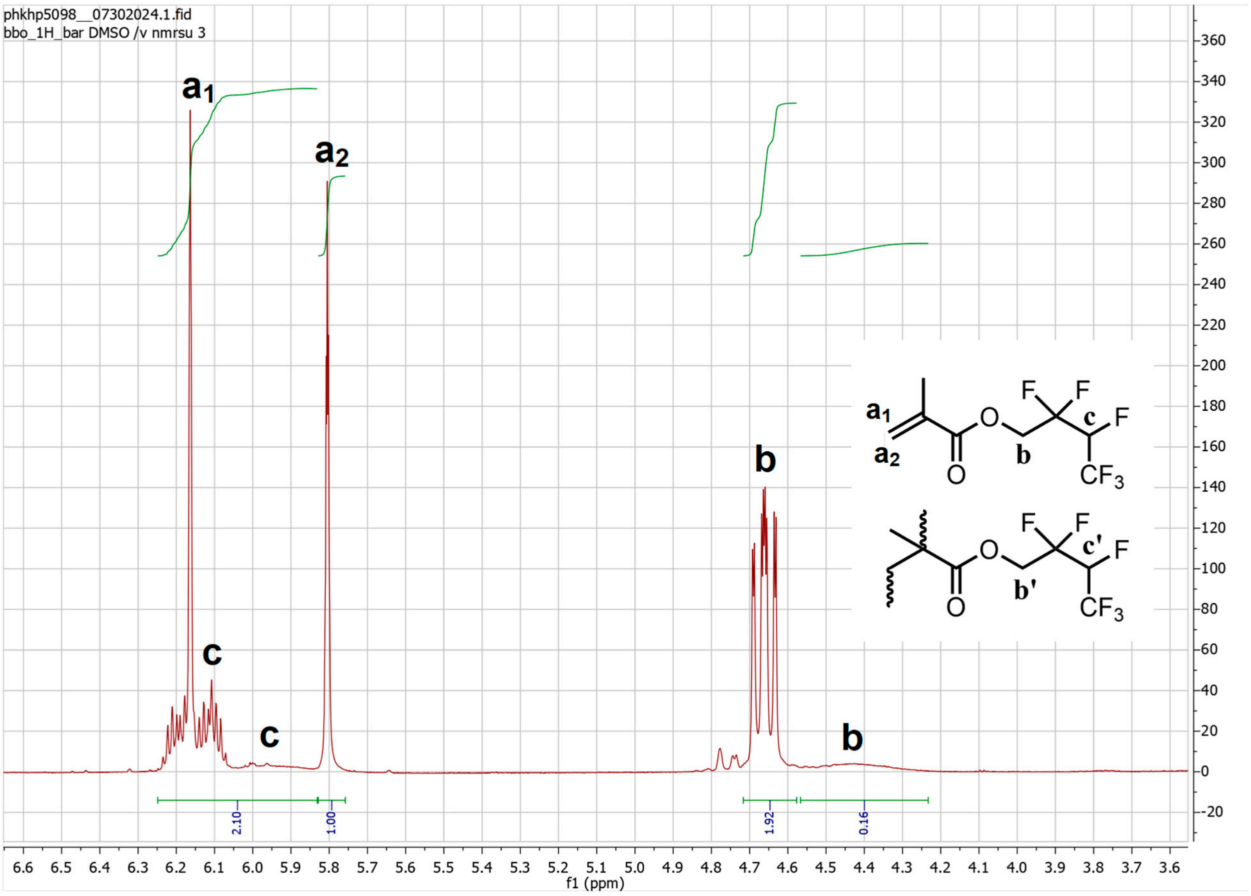

Figure S4. ^{1}H NMR spectrum (DMSO-d^{6}) of initiator-free **FMA** polymerization mixture after being irradiated for blue light for 24 h.

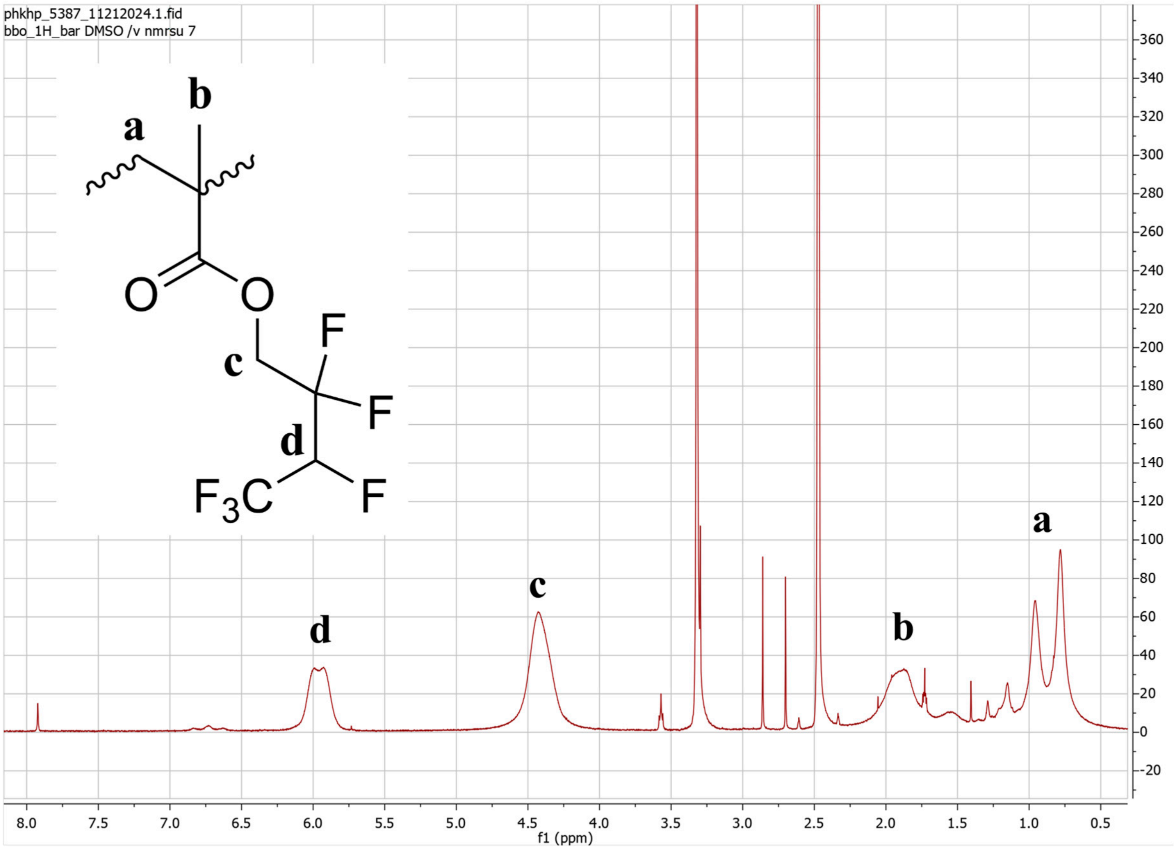

Figure S5. ^{1}H NMR spectrum (DMSO-d^{6}) of **PFMA** reaction mixture (Entry 1, Table S2).

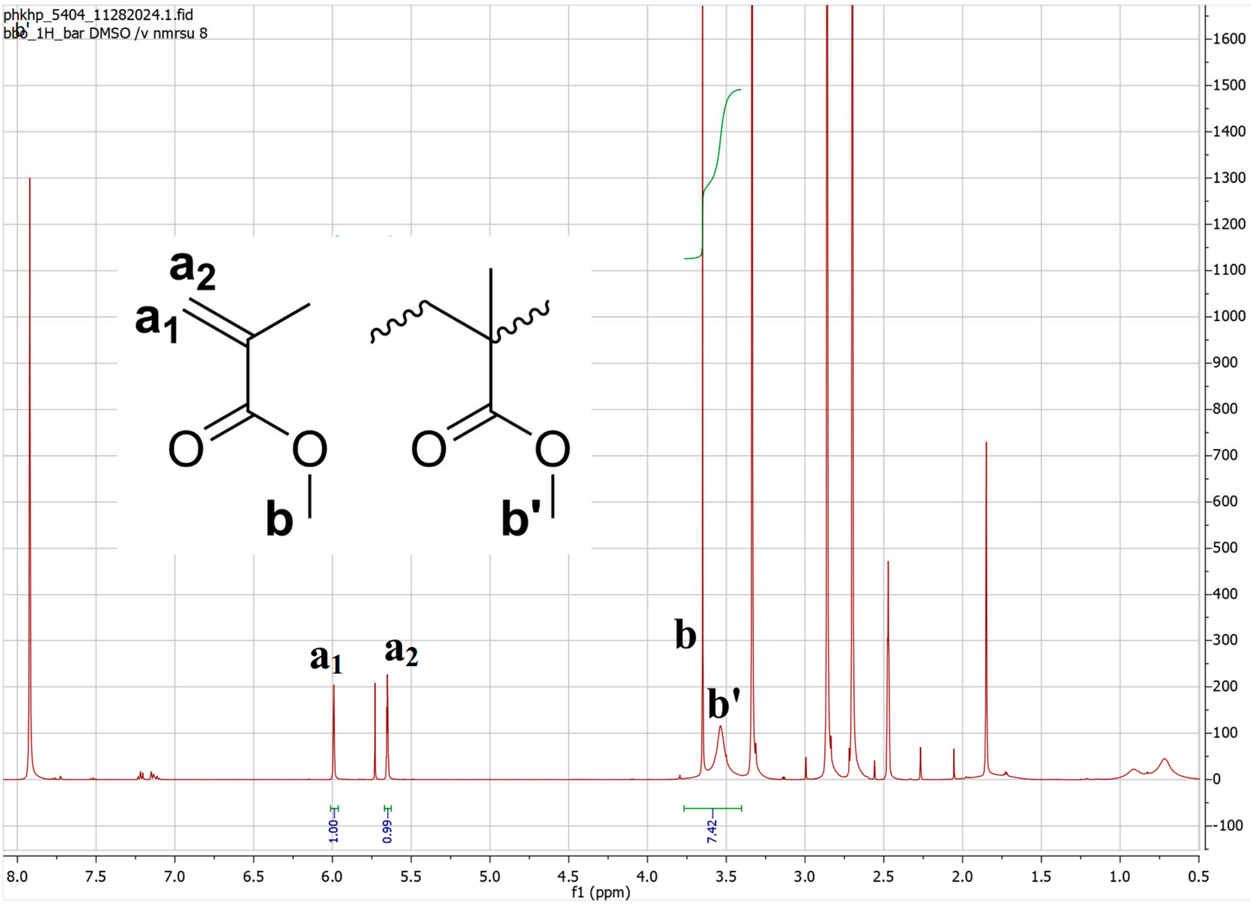

Figure S6. ^{1}H NMR spectrum (DMSO-d^{6}) of MMA polymerization mixture after being irradiated for blue light for 25 h in presence of **PFMA** as C-F initiator (Entry 2, Table S2)

Table S2. Investigation of carbon-fluorine bond activation.

Entry	Monomer	Initiator	PC	Stimulus	Time, h	Conv., %	M_n(SEC), g/mol	Đ
1	**FMA**	AIBN	-	80 °C	28	93[a]	26700	1.58
2	MMA	**PFMA**	Per	435 nm, r.t.	25	61[b]	120400	1.93
					48	93	102400	1.47
3	MMA	-	Per		25	28[b]	249500	1.96
					48	60	306800	2.41
4	MMA	-	-		24	20[a]	874000	1.59

[a] Conversion was determined gravimetrically. [b] Conversion was determined by ^{1}H NMR. Polymerization conditions: V(M) = V(DMF), ω(AIBN) = 3%, $[M]_0/[I^{C\text{-}F}]_0/[per]_0 = 900/9/1$.

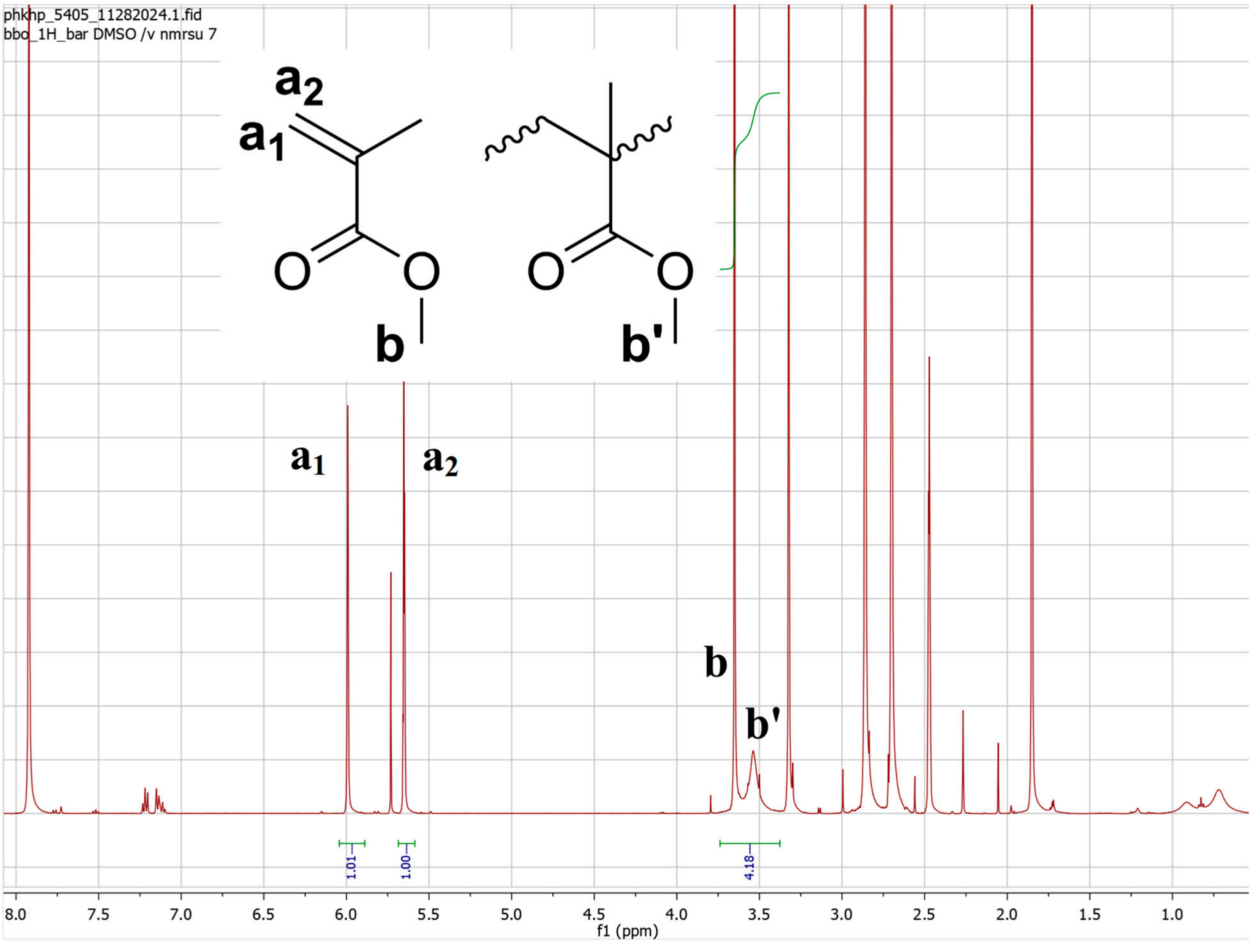

Figure S7. ^{1}H NMR spectrum (DMSO-d^{6}) of initiator-free **MMA** polymerization mixture after being irradiated for blue light for 25 h (Entry 3, Table S2)

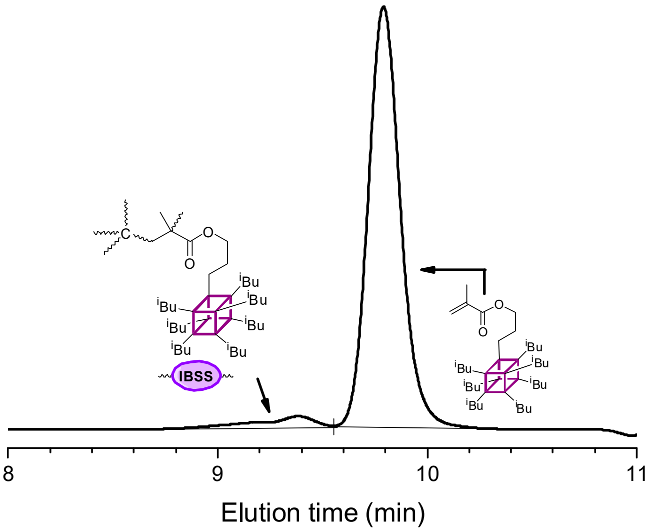

Figure S8. SEC-curves for synthesized **P(IBSS)$_4$**

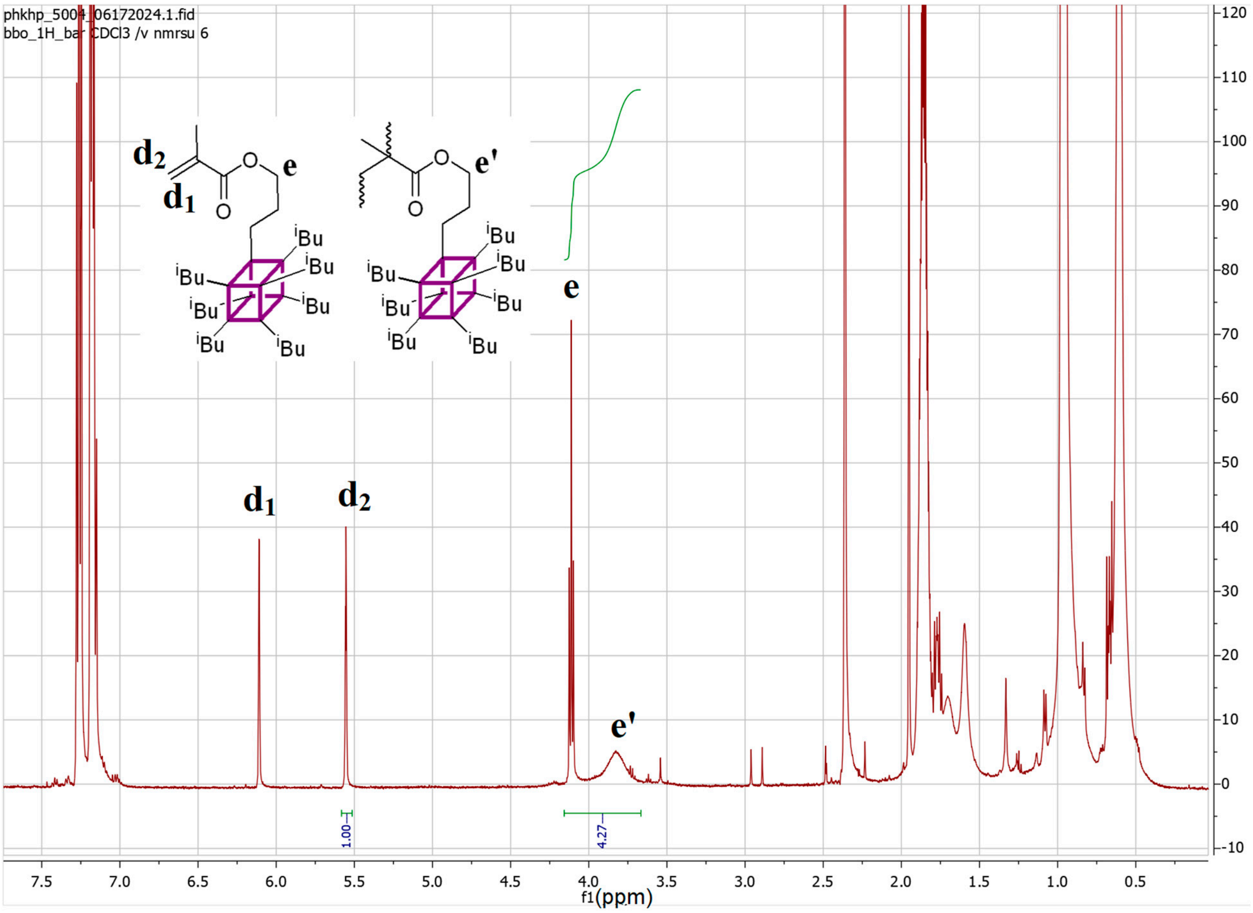

Figure S9. ^{1}H NMR spectrum (DMSO-d^{6}) of **IBSS** polymerization mixture

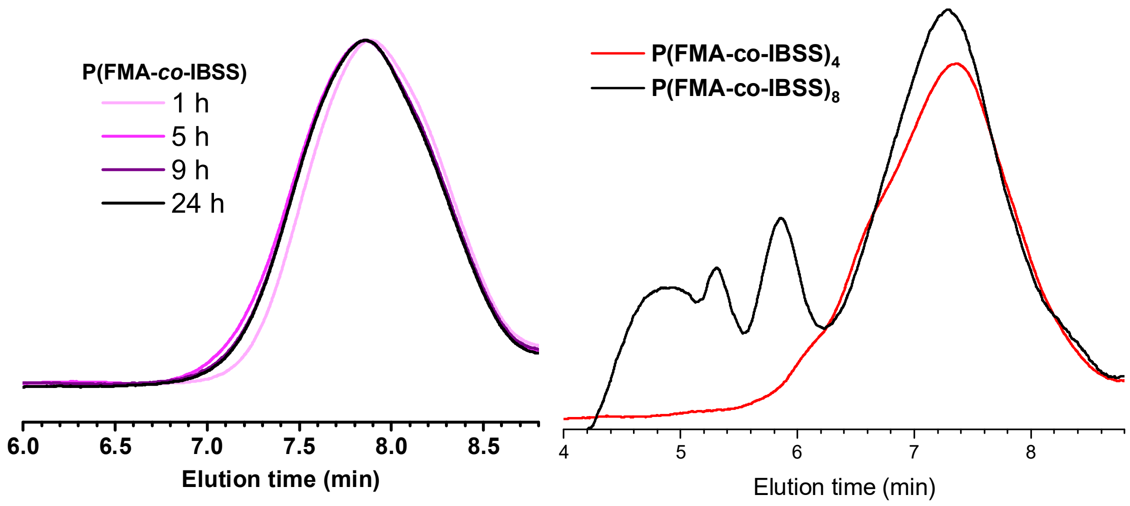

Figure S10. SEC curves for the synthesized random copolymers.

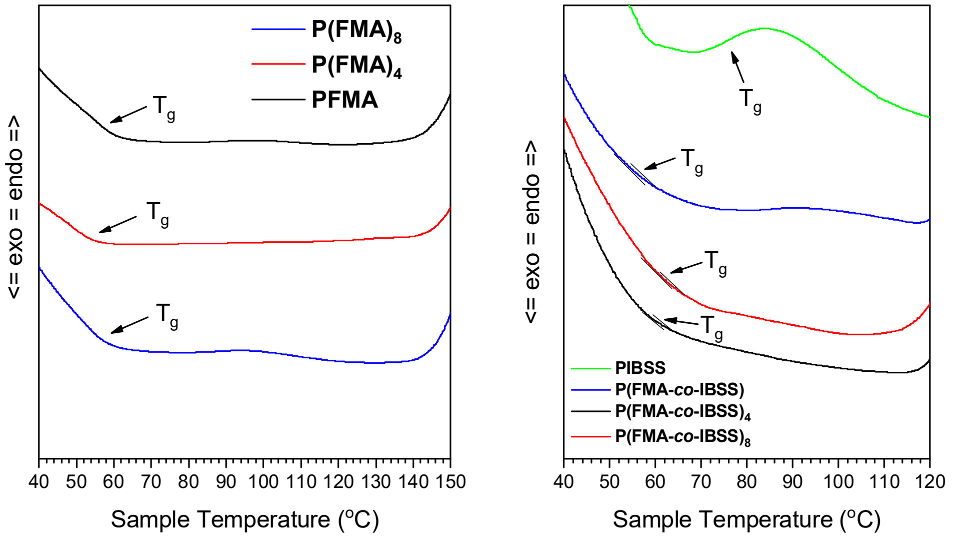

Figure S11. DSC curves of the synthesized (co)polymers

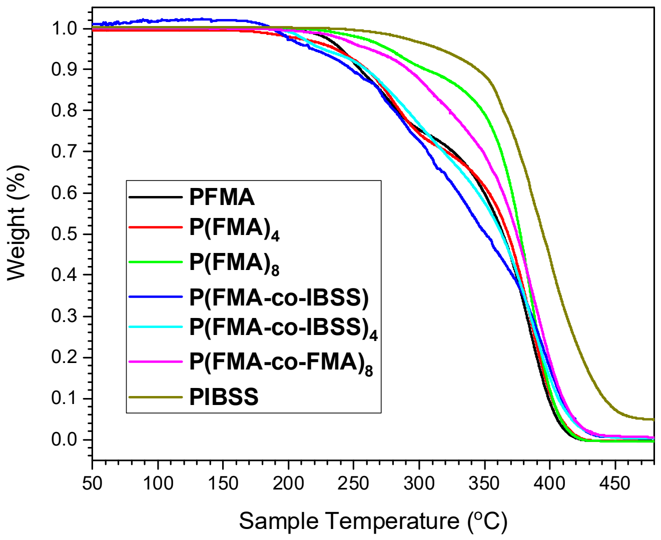

Figure S12. TGA curves of the synthesized (co)polymers

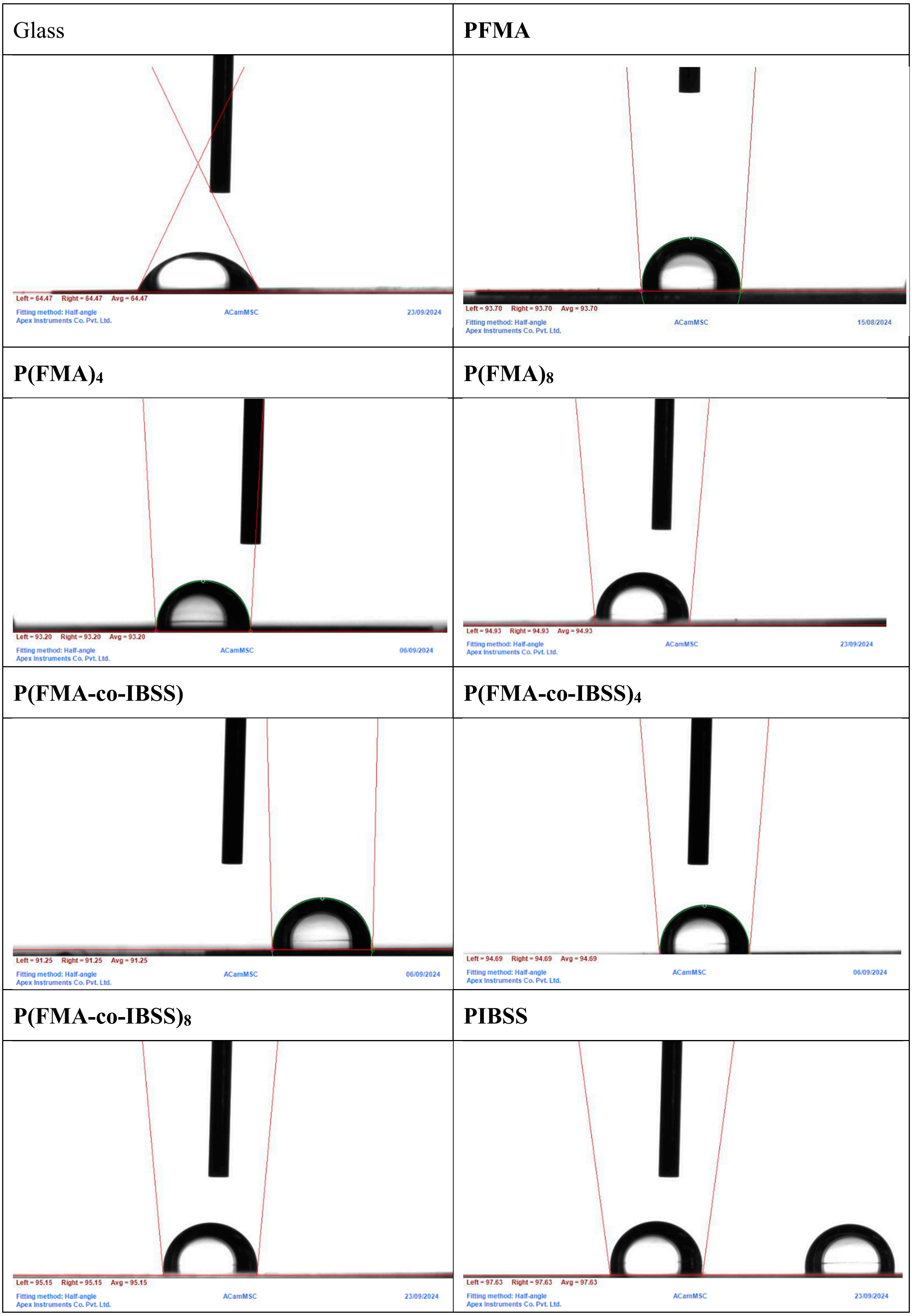

Figure S13. Measurement of contact angles with water (an image of one of the measurements is presented for each polymer film)

Glass	PFMA

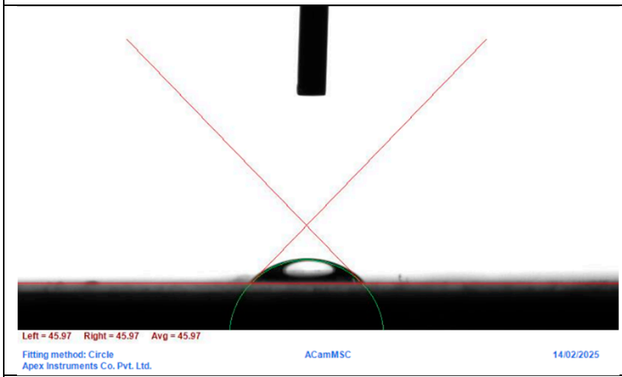

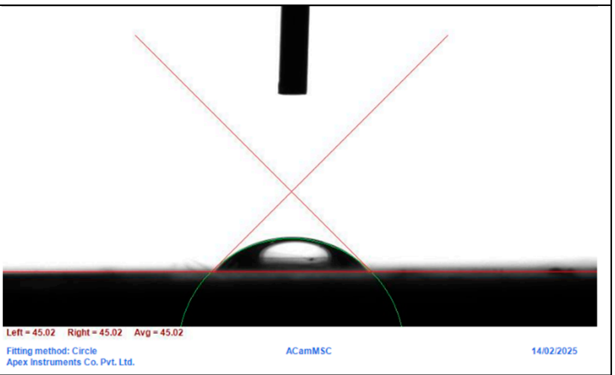

P(FMA)$_4$	P(FMA)$_8$

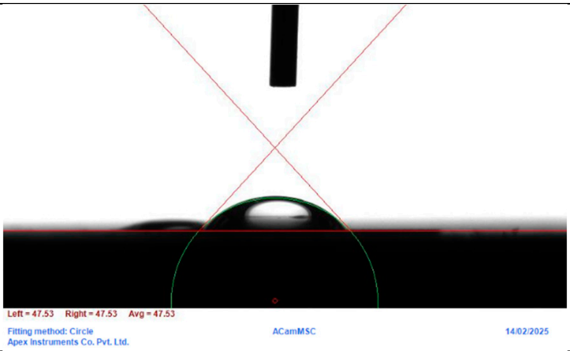

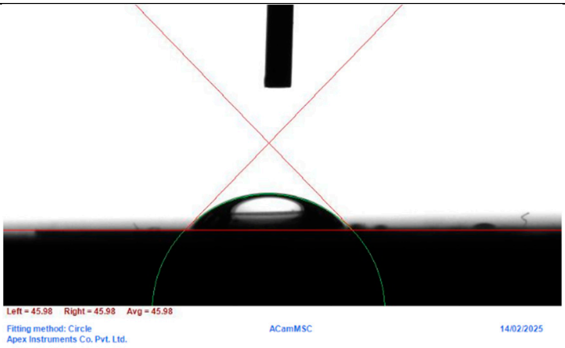

P(FMA-co-IBSS)	P(FMA-co-IBSS)$_4$

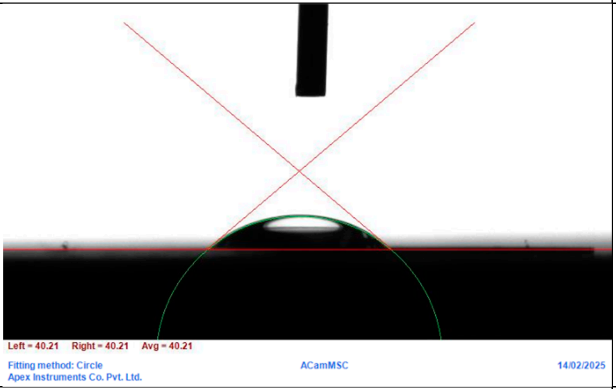

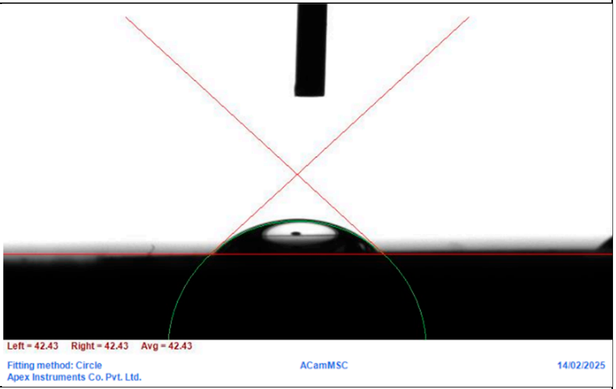

P(FMA-co-IBSS)$_8$	PIBSS

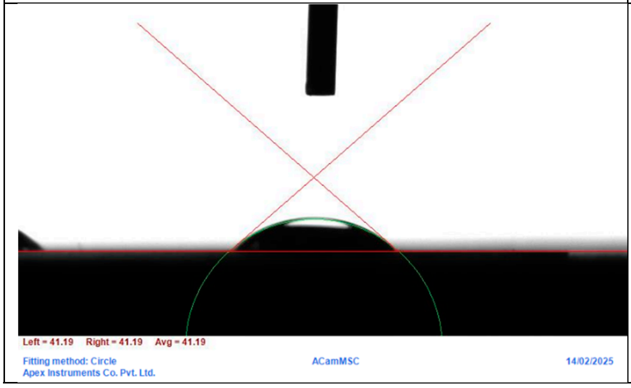

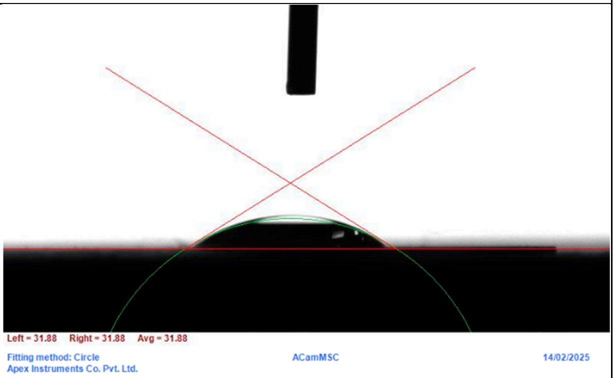

Figure S14. Measurement of contact angles with vegetable oil (an image of one of the measurements is presented for each polymer film)
